# Stage at diagnosis and stage-specific survival of breast cancer in Latin America and the Caribbean: A systematic review and meta-analysis

**DOI:** 10.1371/journal.pone.0224012

**Published:** 2019-10-16

**Authors:** Lívia Lovato Pires de Lemos, Mirian Carvalho de Souza, Daniela Pena Moreira, Paulo Henrique Ribeiro Fernandes Almeida, Brian Godman, Stéphane Verguet, Augusto Afonso Guerra, Mariangela Leal Cherchiglia

**Affiliations:** 1 Programa de Pós-Graduação em Saúde Pública, Faculdade de Medicina, Universidade Federal de Minas Gerais, Belo Horizonte, Minas Gerais, Brazil; 2 SUS Collaborating Centre for Technology Assessment and Excellence in Health, Faculdade de Farmácia, Universidade Federal de Minas Gerais, Belo Horizonte, Minas Gerais, Brazil; 3 Divisão de Pesquisa Populacional, Instituto Nacional de Câncer José Alencar Gomes da Silva, Rio de Janeiro, Rio de Janeiro, Brazil; 4 Programa de Pós-Graduação em Medicamentos e Assistência Farmacêutica, Faculdade de Farmácia, Universidade Federal de Minas Gerais, Belo Horizonte, Minas Gerais, Brazil; 5 Strathclyde Institute of Pharmacy and Biomedical Sciences, Strathclyde University, Glasgow, Scotland; 6 Division of Clinical Pharmacology, Karolinska University Hospital Huddinge, Karolinska Institutet, Huddinge, Sweden; 7 Department of Global Health and Population, Harvard T.H. Chan School of Public Health, Boston, Massachusetts, United States of America; Leibniz Institute for Prevention Research and Epidemiology BIPS, GERMANY

## Abstract

**Background:**

Female breast cancer is the most common cancer in Latin American and Caribbean (LAC) countries and is the leading cause of cancer deaths. The high mortality-to-incidence ratio in the regions is associated with mainly the high proportion of advanced stage diagnosis, and also to inadequate access to health care. In this study we aimed to systematically review the proportion of advanced stage (III-IV) at diagnosis (p_as_) and the five-year stage-specific survival estimates of breast cancer in LAC countries.

**Methods:**

We searched MEDLINE, Embase, and LILACS (Latin American and Caribbean Health Science Literature) to identify studies, in any language, indexed before Nov 5, 2018. We also conducted manual search by reviewing citations of papers found. p_as_ was summarized by random effects model meta-analysis, and meta-regression analysis to identify sources of variation. Stage-specific survival probabilities were described as provided by study authors, as it was not possible to conduct meta-analysis. PROSPERO CRD42017052493.

**Results:**

For p_as_ we included 63 studies, 13 of which population-based, from 22 countries comprising 221,255 women diagnosed from 1966 to 2017. The distribution of patients by stage varied greatly in LAC (p_as_ 40.8%, 95%CI 37.0% to 44.6%; I^2^ = 99%; p<0.0001). The heterogeneity was not explained by any variable included in the meta-regression. There was no difference in p_as_ among the Caribbean (p_as_ 43.0%, 95%CI 33.1% to 53.6%), Central America (p_as_ 47.0%, 95%CI 40.4% to 53.8%) and South America (p_as_ 37.7%, 95%CI 33.1% to 42.5%) regions. For 5-year stage-specific survival we included 37 studies, comprising 28,988 women from ten countries. Seven of these studies were included also for p_as_. Since we were unable to adjust for age, comparability between countries and regions was hampered, and as expected, the results varied widely from study to study.

**Conclusions:**

LAC countries should look to address concerns with early detection and diagnosis of breast cancer, and wherever viable implement screening programs and to provide timely treatment.

## Introduction

In 2018, of the 18 million new cancer cases diagnosed, female breast cancer was the second most frequent, corresponding to 11% of all cancer cases. In Latin America and the Caribbean (LAC) breast cancer was the most common and also the leading cause of cancer related death among women [1]. This pattern was observed previously [2] and is likely to continue in the coming decades. Although the incidence of breast cancer in LAC countries is almost half of that of Europe and North America, the mortality-to-incidence ratio is higher [3].

Despite most LAC countries being classified as upper-middle or high-income by the World Bank [4], social inequality and disparities are is still high in the region [5]. This corroborates the relative high mortality-to-incidence ratio of breast cancer observed, as the relationship between low socioeconomic status and poor breast cancer outcomes is well-established [6]. Among the 11 countries from LAC that contributed to the third global surveillance of trends in cancer survival (CONCORD-3), six reported age-standardized five-year net survival of breast cancer lower than 80% between 2010 and 2014, among them, were populous countries including Brazil and Colombia [7].

The low overall survival estimates are mainly related to the high proportion of women diagnosed with advanced disease, but also to lower access to proper treatment in LAC countries. In 2013, *The Lancet Oncology* Commission identified the following goal for LAC: “Avoid late diagnosis of stage IV cancer to reduce morbidity, mortality, and financial cost”. Suggestions to achieve this goal include optimizing early detection; developing targeted screening programs; implementing clinical early diagnosis programs; and optimizing the treatment of primary cancer [8]. Some studies have compiled the proportions of advanced stage diagnosis [9,10], but, to our knowledge none has assessed this systematically. Stage-specific survival, which may represent an important tool to examine the care each cancer stage is receiving, also has not been studied systematically.

With this review, we intended to systematically assess the distribution of stage at diagnosis of breast cancer in LAC, examining the proportion of advanced disease diagnosis, and possibly the stage-specific survival data from the region. In this way, we hope to provide information for breast cancer control and future guidance to all key stakeholders in the countries in the region.

## Methods

### Search strategy and selection criteria

For this systematic review with meta-analysis, we developed a study protocol (S1 File) following the recommendations of PRISMA guidelines [11] (S1 Table) which was registered in PROSPERO under the number CRD42017052493. There was no funding source for this study. On November 5, 2018, we searched MEDLINE, Embase and Latin American, and Caribbean Health Sciences Literature (LILACS) to identify studies reporting the stage at diagnosis and/or stage-specific survival probability of breast cancer in LAC countries. For this, we used the terms “breast cancer” (Medical Subject Heading (MeSH) and synonyms) and the names of all Latin American and Caribbean countries (as defined by the United Nations) [12], and demonyms (e.g., Argentina OR Argentinian) (S2 File). We conducted manual searches in the reference list of included studies and systematic reviews, PAHO Virtual Health Library regional databases, Scientific Electronic Library Online regional databases and MedCarib. Gray literature was considered for inclusion if no peer-reviewed study was included for the country or region of the country. No restrictions were imposed with respect to the setting of diagnosis or treatment, whether it was private or public, or the language of the publication.

Study selection was conducted in two steps, (i) title and abstract and (ii) full text, in duplicate by two authors (LLPL and PHRFA). Conflicts over the inclusion of potential studies in the review were resolved by consensus between the two authors (LLPL and PHRFA). Rayyan application was used for title and abstract screening (https://rayyan.qcri.org) [13]. Observational studies evaluating women living in LAC countries with confirmed diagnosis of invasive breast cancer were considered eligible. For survival probability, clinical trials were also considered eligible. We excluded studies evaluating: LAC women living in other regions (sometimes referred to *latinas*); patients diagnosed with Paget’s disease or Phyllodes tumor or which gave results including such patients; lactating and pregnant women; and exclusively men. Considering that the incidence of male breast cancer is very low, studies that involve both sexes were not excluded even if results were not presented separately. Studies with a smaller population from the same location/registry of an included study were excluded because of the potential to include repeated patients. Multi-country studies not reporting results by country were excluded. We also excluded studies reporting only survival probability of early stages that included *in situ* cases and studies that reported stage at diagnosis only as aggregate categories including *in situ* stage (e.g.; early stage: 0-IIa). For the survival probability outcome we excluded studies reporting only hazard ratios, and for stage at diagnosis we excluded studies evaluating specific disease stages.

### Data extraction and quality assessment

Data extraction and quality assessment were performed in duplicate by two authors (LLPL and DPM) with discordances resolved by consensus among them. We used a specially designed spreadsheet to collect information regarding: country; study design; study setting (name of studied health services; name of population-based registry); if the study included stage at diagnosis or survival probability outcomes or both; number of included patients; year of diagnosis; age at diagnosis; menopausal status; histology type; tumor grade; hormonal receptor status; HER-2 status; molecular subtype; and race. For studies included for stage at diagnosis we collected the staging criteria; the number of patients who presented in the different stages as given by the study authors, i.e., using the most disaggregated Tumor, Lymph Node, and Metastasis (TNM) staging (Ia, Ib, …, IV), TNM or Manchester stages I, II, III and IV, TNM or Manchester aggregated stages (I-II, III-IV, I-IIa, IIb-IV), or SEER staging (localized, regional, distant). For studies included for survival probability we collected the starting date of survival analysis; if overall survival, disease specific survival or both were provided; the survival probability with standard error or confidence interval; the number of individuals at risk and number of events; the method for survival analysis (e.g., Kaplan Meier), and the information regarding staging classification as with the stage at diagnosis outcome.

For quality assessment of studies reporting stage at diagnosis we used the tool developed by Elm *et al*. [14] and adapted by Jedy-Agba *et al*. [15]. For quality assessment of the studies reporting survival probability we adapted this tool accounting for the potential sources of bias in longitudinal studies exemplified by Chubak *et al*. [16] and items of The Newcastle-Ottawa Scale (NOS) for assessing the quality of non-randomized studies in meta-analyses [17]. In both cases, we evaluated three domains: selection bias, information bias, and other factors related to stage at diagnosis/survival analysis, such as age and tumor grade, with more value given to selection and information bias items. The quality score ranged from 0–28 (low to high) in both scales (S2 and S3 Tables). If a study reported both stage at diagnosis and survival rate, it was evaluated separately in each tool.

### Data analysis

For stage at diagnosis, we used R package “meta” to pool the primary outcome with a random effects model (https://github.com/guido-s/meta http://meta-analysis-with-r.org) [18,19]. The outcome was the percentage (p_as_) of breast cancer diagnosed in stages III-IV which was calculated as p_as_ = (n_as_/n)*100, where n_as_ is the number of patients presented at advanced stages and n is the number of staged patients. We considered between-study heterogeneity present when the P value of the Cochran’s Q test was <0.1 and I^2^ statistic was >50%. To examine potential sources of heterogeneity, study-specific estimates were stratified by relevant variables and a meta-regression analysis was performed to identify correlates of percentage of advanced stage disease. Study-level determinants of advanced stage disease are expressed as absolute differences (AD) in the percentage of patients with advanced stage disease (p_as_). Potential publication bias was estimated with the Egger’s test.

The primary outcome for stage-specific survival was the five-year all-cause survival of patients diagnosed with invasive breast cancer. Secondary outcomes were cause-specific survival probability and global survival at any time. Survival probability outcomes may be from any date (diagnosis, start of treatment, first consultation, etc.). Since the large majority of studies did not report the number of patients at risk and the number of events, a requirement for survival probability meta-analysis, we were unable to perform meta-analysis and meta-regression for this outcome. Consequently, the survival probability outcomes were described as the study authors provided it (percentage with or without variance).

## Results

**Fig 1:**
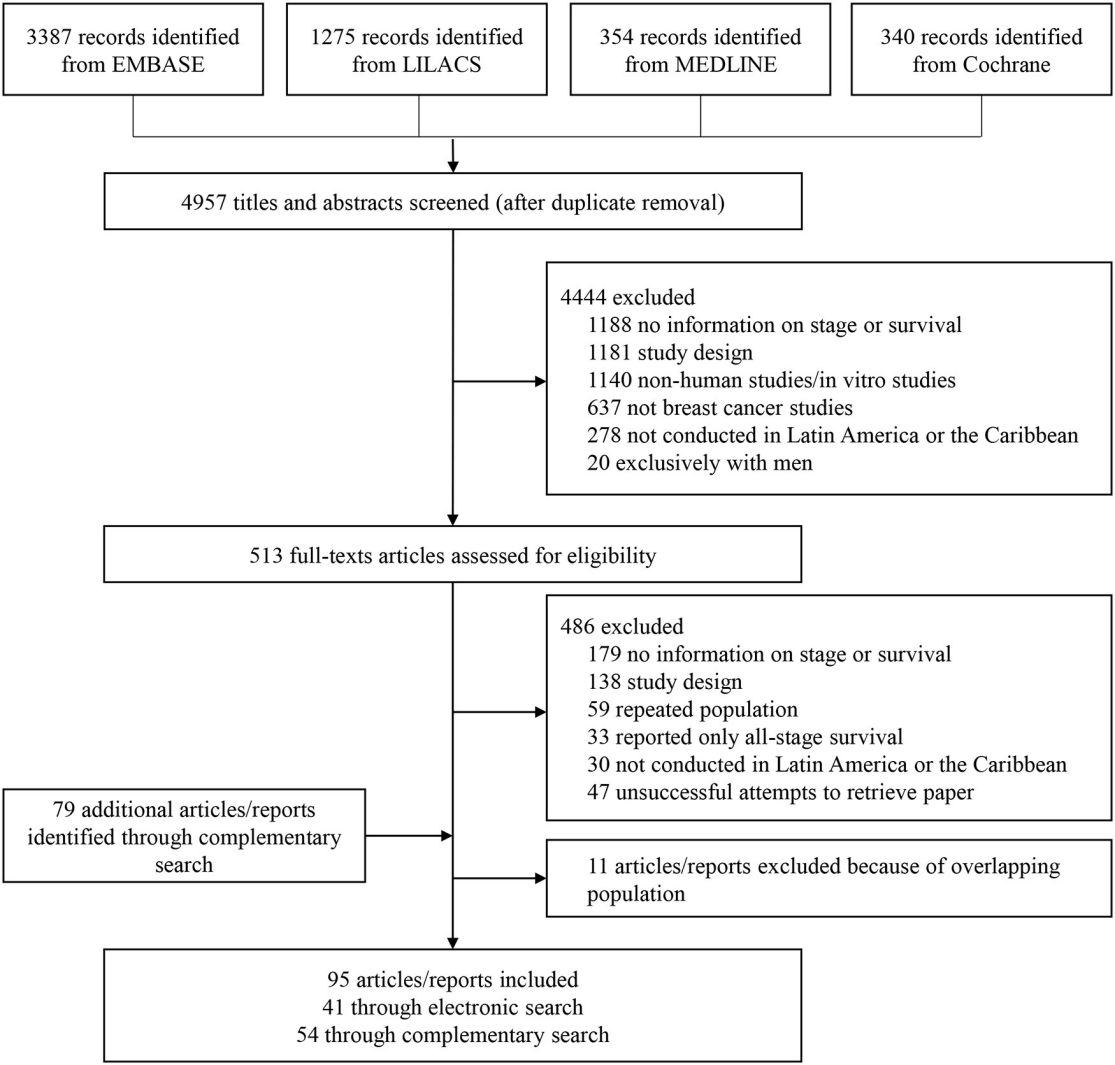
Study selection flowchart.

After duplicates removal, 4,957 records had their titles and abstracts assessed, resulting in 513 full-text studies that were assessed for eligibility. The complementary search yielded 79 documents that were assessed for eligibility. We finally included 95 studies (Fig 1), 46 studies assessed breast cancer stage-specific survival in 12 countries, and 63 assessed breast cancer stage at diagnosis in 22 countries (14 studies assessed both). For both outcomes, most studies consisted of consecutive case series, conducted in public facilities with individuals between 40 and 59 years old diagnosed between 2000 and 2009. In the five studies that included men, male population represented 0.3% to 0.7% of the sample [20–24]. For countries we did not find peer-reviewed studies we searched for epidemiological/registry reports and Ecuador was the only country we could identify and include reports [25–27]. We also included a cancer hospital registry report from a region of Peru for which we did not include a peer-reviewed study [28]. As for the quality score, in studies included for the outcome of stage at diagnosis, most patients participated on studies from intermediate to low quality scores (15.5 to 18.8). (Table 1 and S2 Table). In studies included for survival probability, most patients participated on high scoring studies (>20.5) (Table 1 and S3 Table). Both scores ranged from 0 to 28. Study references are given in the Supporting Information (S3 File).

**Table 1:** Study characteristics.

	Survival			Stage at presentation		
	Studies	Patients with breast cancer	Patients with known breast cancer stage (%)	Studies	Patients with breast cancer	Patients with known breast cancer stage (%)
Total	46	34,282	30,861 (90.0)	63	263,515	221,255 (84.0)
Region and country						
Caribbean						
Bahamas				1	270	134 (46.6)
Barbados				1	222	222 (100.0)
Cuba	6	5,159	4,761 (92.3)	6	3,998	3,580 (89.5)
Haiti	1	525	127 (24.2)	1	525	445 (84.8)
Jamaica				1	199	184 (92.5)
Puerto Rico				1	985	867 (88.0)
Trinidad and Tobago				2	4,130	3,458 (83.7)
Central America						
Costa Rica	2	2,683	2,326 (86.7)	1	2,462	2,105 (85.5)
Honduras				1	685	653 (95.3)
Mexico	9	13,198	11,854 (89.8)	10	14,815	13,978 (94.4)
South America						
Argentina	3	1,882	1,828 (97.1)	6	8,454	7,344 (86.9)
Brazil	8	4,030	3,769 (93.5)	3	188,645	154,889 (82.1)
Chile	3	1,447	1,318 (91.1)	2	23,357	21,477 (92.0)
Colombia	4	1,805	1,662 (92.1)	11	6,038	5,405 (89.5)
Ecuador	1	21	21 (100)	3	2,438	2,079 (85.3)
French Guiana				1	269	239 (88.8)
Guyana				1	499	445 (89.2)
Paraguay				1	80	80 (100.0)
Peru	2	354	147 (41.5)	5	4,178	2,514 (61.0)
Suriname*				1	419	351 (83.8)
Uruguay	1	1,311	1,185 (90.4)	2	222	216 (97.3)
Venezuela	6	1,867	1,863 (99.8)	2	625	590 (94.4)
Study design (sampling)						
Convenience	4	7,177	6,422 (89.5)	2	1,161	1,097 (94.5)
Consecutive	31	22,619	20,195 (89.3)	47	249,196	209,245 (84.0)
Population-based	3	1,640	1,602 (97.7)	13	13,099	10,869 (83.0)
Unclear	8	2,846	2,642 (92.8)	1	59	54 (91.5)
Type of facility						
Private†	10	6,510	5,635 (86.6)	7	3,925	3,520 (89.7)
Public and Private	2	3,207	2,831 (88.3)	11	11,685	10,016 (85.7)
Public	30	22,085	20,081 (90.9)	37	237,839	199,006 (83.7)
Not reported in original study	4	2,480	2,314 (93.3)	8	10,066	8,713 (86.6)
Age at diagnosis (years)‡						
<40 years				1	107	107 (100.0)
≥40 to <60 years	41	30,072	26,781 (89.1)	51	183,937	155,502 (84.5)
≥60 years	3	1,703	1,573 (92.4)	5	962	766 (79.6)
Not reported in original study	2	2,507	2,507 (100)	6	78,509	64,880 (82.6)
Year of diagnosis¶						
Before 1999	23	15,987	14,555 (91.0)	12	60,528	49,533 (81.8)
2000–2009	21	17,278	15,687 (90.8)	44	194,787	164,689 (84.5)
2010 or after	1	525	127 (24.2)	7	8,200	7,033 (85.8)
Not reported in original study	1	492	492 (100)			
Staging methods						
Clinical and imaging	8	8,213	7,553 (92.0)	7	6,002	5,749 (95.8)
Clinical only	3	662	662 (100)			
Not reported in original study||	35	25,407	22,646 (89.1)	53	257,513	215,506 (83.7)
Staging classification						
TNM	39	25,249	22,723 (90.0)	52	250,429	219,990 (83.9)
Manchester/SEER/NCCN	3	7,006	6,252 (89.2)	4	5,979	5,107 (85.4)
Not reported in original study||	4	2,027	1,886 (93.0)	7	7,107	6,158 (86.6)
Study quality scores††						
≤15 (lowest quality)	7	6,936	6,550 (94.4)	9	8,833	6,587 (74.6)
15.5–18.5	9	5,232	5,030 (96.1)	24	228,308	191,045 (83.7)
19–20.5	11	7,056	6,491 (92.0)	18	18,737	16,402 (87.5)
>20.5 (highest quality)	19	15,058	12,790 (84.9)	12	76,37	7,221 (94.6)

Data are n or n (%). Study references are given in the Supporting Information (S3 File). TNM, Tumor, Lymph Node, and Metastasis staging system; SEER, Surveillance, Epidemiology, and End Results Summary Stage (localized, regional, distant).

* SUR-van Leeuwaarde (2011) provided tumor size, T3/4 were considered as a proxy for stages III/IV.

^†^ Includes non-profit organizations.

^‡^ Mean or median age at breast cancer diagnosis. If only age categories were provided, mean or median age was estimated from the midpoint and the reported number in each category.

^¶^ Middle year of the time interval of patient recruitment or diagnosis.

^††^ Categories represent quartiles of the overall score distribution.

**Fig 2:**
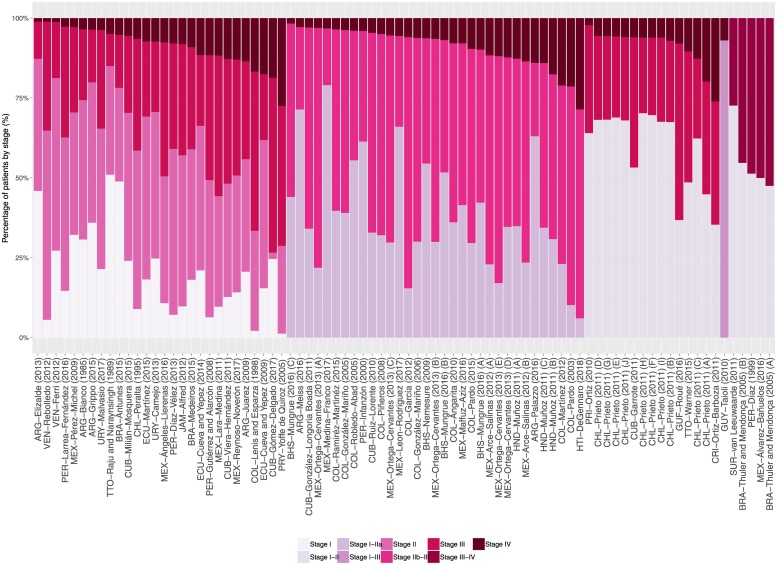
Study-specific percentage of patients by stage at presentation. Percentage of T3/T4 cancers were used as proxy of stages III/IV for SUR-van Leeuwaarde (2011). Localized, regional and distant disease were considered as stages I-II, III and IV for CRI-Ortiz-Barboza (2011), CUB-Garrote (2011), and PRI-Ortiz (2010). Recruitment or diagnosis years: BHS-Mungrue (2016) A→C = 2009→2011; BRA-Thuler and Mendonça (2005) A = 1990–1994 B = 1995–2002; CHL-Prieto (2011) A→J = 2000→2011; HND-Muñoz (2011) A = 1999 B = 2000–2004 C = 2005–2009; MEX-Ortega-Cervantes (2013) A→E = 2006→2010; MEX-Arce-Salinas (2012) A = 2008 B = 2009. Study references are given in the Supporting Information (S3 File).

**Fig 3:**
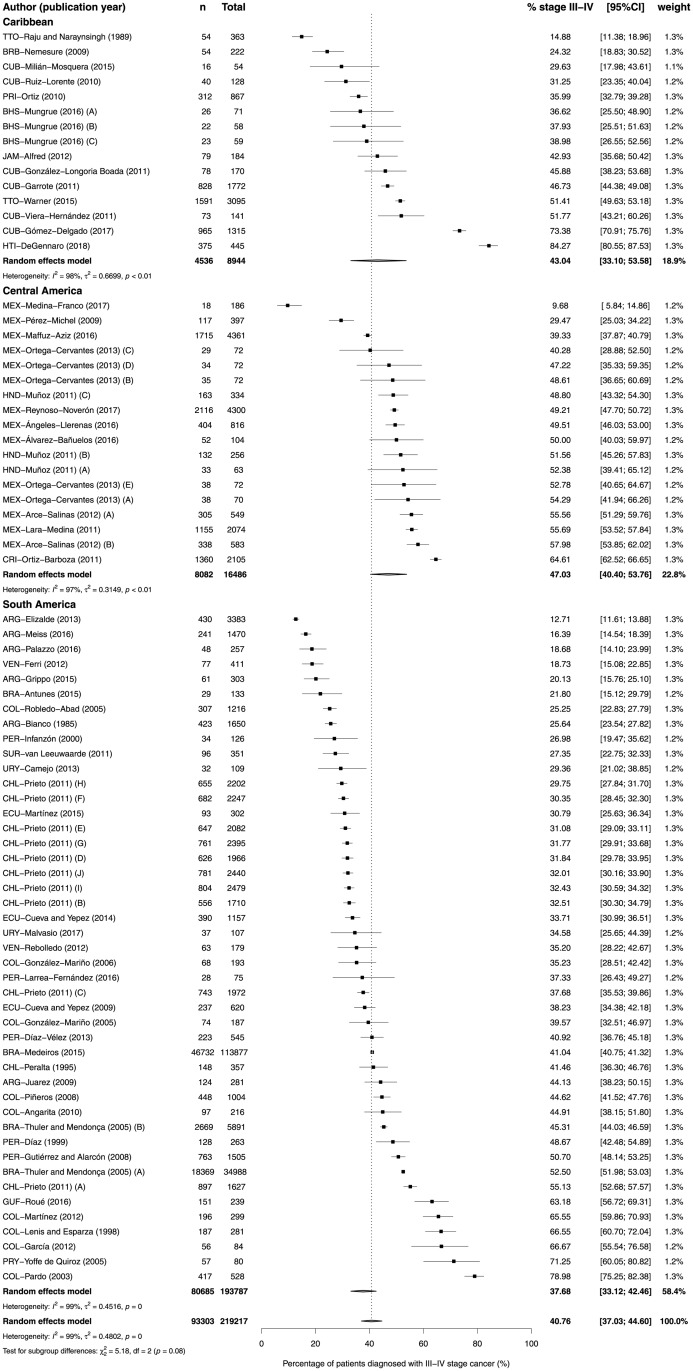
Forest-plot of percentage of stage III-IV breast cancer at diagnosis, by region of Latin America and Caribbean. Percentage of T3/T4 cancers were used as proxy of stages III/IV for SUR-van Leeuwaarde (2011). Regional and distant disease were considered as stages III and IV for CRI-Ortiz-Barboza (2011), CUB-Garrote (2011), and PRI-Ortiz (2010). The study GUY-Taioli (2010) was not included because it provided the proportion of patients diagnosed with stages I-III (93%) and stage IV (7%). Recruitment or diagnosis years: BHS-Mungrue (2016) A→C = 2009→2011; BRA-Thuler and Mendonça (2005) A = 1990–1994 B = 1995–2002; CHL-Prieto (2011) A→J = 2000→2011; HND-Muñoz (2011) A = 1999 B = 2000–2004 C = 2005–2009; MEX-Ortega-Cervantes (2013) A→E = 2006→2010; MEX-Arce-Salinas (2012) A = 2008 B = 2009. Study references are given in the Supporting Information (S3 File).

The 63 studies assessing the stage at diagnosis comprised 263,515 patients, 84.0% with known stage at diagnosis. Sample sizes ranged from 59 to 137,593 (median 345). Four studies used a staging system other than TNM and in seven studies the staging system was not reported (Table 1 and S4 Table). The distribution of patients by stage varied greatly in LAC (S5 Table). In studies that reported stage IV percentage, this varied from approximately 1% in one study from Argentina with 4041 women diagnosed between 2010 and 2012 and one study from Venezuela with 179 women diagnosed between 1999 and 2007, to 26% in one study from Costa Rica with 2462 women diagnosed between 1995 and 2000 and 29% in one study from Haiti with 525 women diagnosed between 2013 and 2017. This study reported the highest proportion of stage III-IV diagnosis, 84.3%; the lowest proportion (9.7%) was observed in one study with 230 women diagnosed between 2001 and 2016 in Mexico (Fig 2). Consequently, there was considerable heterogeneity in the proportion of patients diagnosed with stage III-IV (p_as_ 40.8%, 95%CI 37.0% to 44.6%; I^2^ = 99%; p<0.0001) (Fig 3).

**Fig 4:**
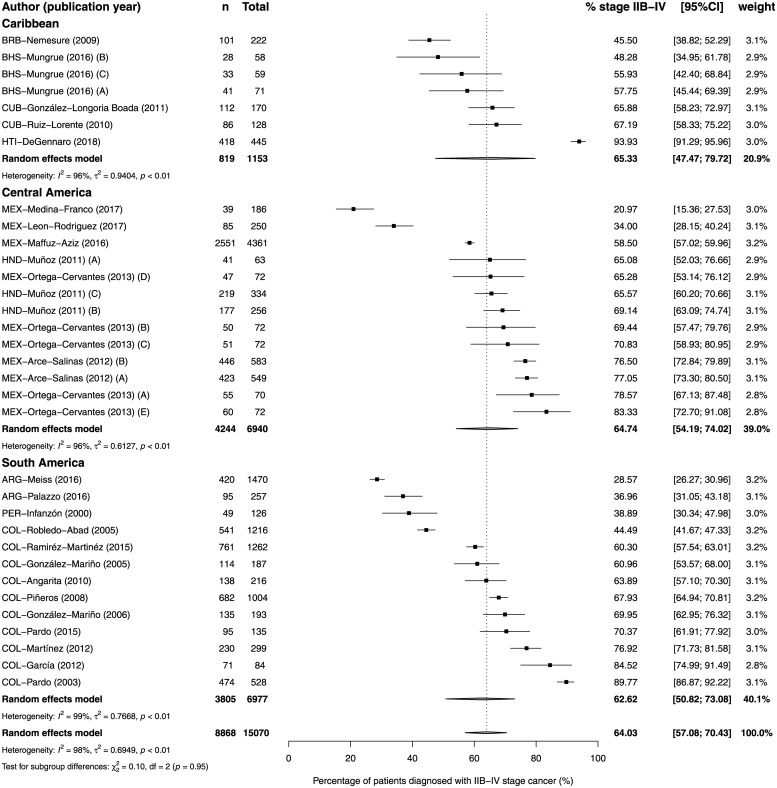
Forest-plot of percentage of stage IIb-IV breast cancer at diagnosis, by region of Latin America and Caribbean. Recruitment or diagnosis years: BHS-Mungrue (2016) A→C = 2009→2011; HND-Muñoz (2011) A = 1999 B = 2000–2004 C = 2005–2009; MEX-Ortega-Cervantes (2013) A→E = 2006→2010; MEX-Arce-Salinas (2012) A = 2008 B = 2009. Study references are given in the Supporting Information (S3 File).

There was no difference between regions, however there was a tendency of lower proportion of patients diagnosed with stage III-IV in South America (p_as_ 37.7%, 95%CI 33.1% to 42.5%; I^2^ = 99%; p<0.0001) and the highest in the Caribbean (p_as_ 43.0%, 95%CI 33.1% to 53.6%; I^2^ = 98%; p<0.01) (Fig 3). Publication bias was difficult to analyze due to the high heterogeneity (S1 Fig). As a post-hoc analysis, we conducted a meta-analysis including patients diagnosed with stage IIb. The overall estimate of the proportion of patients diagnosed with stage IIb-IV was 64.0% (95%CI 57.0% to 70.4%; I^2^ = 98%; p<0.01). There was no difference between regions and no tendency was observed (Fig 4).

Adjusted meta-regression did not reveal any association between the proportion of stage III-IV diagnosis and any of the available variables. The adjusted meta-regression revealed that consecutive case series presented a higher proportion of stage IIb-IV diagnosis than population-based studies (Absolute Difference, AD 49.5, 95%CI 15.5% to 83.5%). Studies conducted in private settings, and studies conducted with older patients (≥60 years old), presented a borderline lower proportion of stage IIb-IV diagnosis, when compared to, respectively, studies conducted in private settings and studies conducted with patients between 40 and 59 years of age (Table 2).

**Table 2:** Meta-regression results: Analysis of predictors of advanced stages breast cancer diagnosis.

Variables	Stages III-IV					Stages IIb-IV*				
	Patients, n	Unadjusted analysis		Adjusted analysis		Patients, n	Unadjusted analysis		Adjusted analysis	
		AD (%)	95% CI	AD (%)	95% CI		AD (%)	95% CI	AD (%)	95% CI
**Region**										
Caribbean	8944	0 (Ref)		0 (Ref)		1153	0 (Ref)		0 (Ref)	
Central America	16486	4.2	-6.5 to 14.8	0.0003	-14.7 to 14.7	6940	1.5	-15.3 to 18.4	-20.3	-50.3 to 9.7
South America	193787	-4.9	-13.9 to 4.1	-9.8	-24.0 to 4.5	6977	-1.5	-18.3 to 15.4	-16.4	-47.2 to 14.5
**Study type (sampling)**										
Population-based	10478	0 (Ref)		0 (Ref)		837	0 (Ref)		0 (Ref)	
Consecutive	207588	1.9	-7.4 to 11.1	11.3	-3.9 to 26.4	14233	13.2	-2.2 to 28.7	**49.5**	**15.5 to 83.5**
Convenience	1097	6.9	-16.4 to 30.2	8.3	-19.1 to 35.7	0				
Unclear	54	-10.3	-44.1 to 23.4	-26.8	-65.4 to 11.8	0				
**Type of provider**										
Public	198675	0 (Ref)		0 (Ref)		8168	0 (Ref)		0 (Ref)	
Private†	3520	-7.9	-19.9 to 4.2	-11.1	-24.3 to 2.1	2063	-9.7	30.9 to -28.3	**-22.4**	**-44.3 to -0.5**
Public and private	10016	-6.8	-16.8 to 3.2	4.0	-12.4 to 20.4	257	-28.7	-63.7 to 6.3	75.1	-5.1 to 55.2
Not reported in original study	7006	-9.3	-20.8 to 2.2	-13.1	-26.6 to 0.4	4582	-4.9	-19.8 to 10.1	-4.5	-20.9 to 11.9
**Age at diagnosis (years)** ‡										
<40	107	0 (Ref)		0 (Ref)		0				
≥40 to <60	153464	8.6	-22.7 to 40.0	16.9	-19.3 to 53.0	14159	0 (Ref)		0 (Ref)	
≥60	766	-5.0	-39.0 to 29.0	-1.0	-38.7 to 36.7	383	-25.3	-48.7 to -1.9	**-45.8**	**-91.4 to -0.3**
Not reported in original study	64880	5.0	-27.0 to 37.0	17.5	-20.5 to 55.6	528	26.6	-5.3 to 58.5	17.7	-16.6 to 52.0
**Year of diagnosis (year)** ¶										
Before 1999	49533	0 (Ref)		0 (Ref)		1512	0 (Ref)		0 (Ref)	
2000–2009	162651	1.1	-8.3 to 10.5	-1.0	-11.74 to 9.8	11386	15.3	-5.4 to 36.0	-12.7	-41.9 to 16.6
2010 or after	7033	-2.9	-17.4 to 11.5	-2.7	-20.2 to 14.7	2172	3.6	-24.0 to 31.1	-27.5	-69.1 to 14.1
**Staging classification**										
TNM	208397	0 (Ref)		0 (Ref)		15070				
Other (Manchester, SEER, NCCN)	5107	-0.001	-15.4 to 15.4	-10.4	-30.2 to 9.4	0				
Not reported in original study	5713	10.9	-2.0 to 23.9	-7.0	-25.2 to 11.2	0				
**Staging methods**										
Clinical and imaging	5803	0 (Ref)		0 (Ref)		633	0 (Ref)		0 (Ref)	
Clinical	0					0				
Unclear	213414	-6.2	-17.1 to 4.8	-2.1	-22.5 to 18.4	14437	-2.7	-21.8 to 16.4	-22.8	-52.3 to 6.7
**Study quality scores** ||										
>20.5 (highest quality)	7275	0 (Ref)		0 (Ref)		890	0 (Ref)		0 (Ref)	
19–20.5	16152	-3.5	-14.2 to 7.2	-0.2	-18.7 to 18.4	4568	1.1	-18.2 to 30.4	-4.2	-19.2 to 10.8
15.5–18.5	189648	-7.5	-17.0 to 2.1	-10.2	-27.9 to 7.4	9612	6.0	-12.2 to 24.3		
≤15 (lowest quality)	6142	3.8	-9.6 to 17.3	12.2	-11.5 to 36.0					

T3/4 were considered as a proxy for stages III/IV. Regional and distant diseases were considered as stages III and IV, respectively. Inoperable locally advanced disease was considered as stage III. AD, Absolute difference; CI, Confidence Interval; TNM, Tumour, Lymph Node, and Metastasis staging system; SEER, Surveillance, Epidemiology, and End Results Summary Stage; NCCN, National Comprehensive Cancer Network classification.

*Post-hoc analysis.

^†^ Includes non-profit organizations.

^‡^ Mean or median age at breast cancer diagnosis. If only age categories were provided, mean or median age was estimated from the midpoint and the reported number in each category.

^¶^ Middle year of the time interval of patient recruitment or diagnosis.

^||^ Categories represent quartiles of the overall score distribution.

The 46 studies assessing survival probability comprised 34,282 patients diagnosed from 1966 to 2017, 90.0% with known disease stage. Sample sizes ranged from 21 to 4,902 (median 345). Thirty-five studies used the Kaplan-Meier method to estimate survival probability, nine studies used the actuarial method and one study from Cuba did not report the survival analysis method [29]. Thirty-seven studies considered deaths from any cause and nine considered disease-related deaths. Most included studies (67%) were hospital-based studies in which consecutive patients were followed-up for a determined period of time, hence studies populations and settings varied greatly. Only four studies were population-based, three from Cuba [23,30,31] and one from Costa Rica [22]. For 5-year stage-specific survival we included 37 studies, comprising 28,988 women from ten countries. Seven of these studies were included also for p_as_. Study-specific details and results are given in the Supporting Information (S6 and S7 Tables).

## Discussion

In this systematic review, we used two markers, the proportion of advanced disease at diagnosis and stage-specific survival, in an attempt to characterize the extent of breast cancer control in LAC countries. With data from 221,255 women from 22 countries diagnosed from 1966 to 2017, we revealed that in these regions nearly 41% women were diagnosed in stages III-IV. The marked heterogeneity of the meta-analysis was not explained by any variable included in the meta-regression. The post hoc analysis with data from 15,070 women from nine countries revealed that 64% were diagnosed in stages IIb-IV. The high heterogeneity in this analysis was explained by the type of study, with studies that used consecutive sampling presenting a higher proportion of late-stage diagnosis than population-based studies. Only four population-based studies with 837 women were included, and of those, two were the single representatives of their countries (Barbados [32] and Bahamas [33]), and the other two were one of two studies included from Argentina [34] and Cuba [30]. Other variables likely to be related to the stage at diagnosis, such as school years, socioeconomic status and race, could not be evaluated because few studies reported them to allow comparability. Low economic status has been related to late stage diagnosis in low- and middle-income [35] and high-income countries [36].

The high percentage of diagnosis in advanced stages in LAC contrasts sharply to the proportions of 8.3% to 23.5% of advanced stage (III-IV) diagnosis among women of Western European countries [37]. This points out to the low coverage of screening and early detection practices in the region. According to the World Health Organization, from 30 LAC countries that responded to the Cancer Country Profile survey in 2014, 19 reported having established cancer control programs or strategies, and of those, 18 stated that clinical breast examination is generally available at the public primary health care level, and 10 reported that mammography was available as well [38]. In this review, only one study assessed the effect of a breast cancer screening program. Maffuz-Aziz *et al*. [39] showed that 83% of women from a screening program from Mexico City were diagnosed with stages 0-IIA versus 36% of women who did not participate on the program. In Mexico, national health surveys revealed low coverage of annual clinical breast exam, no higher than 55%, and the very low annual mammography coverage of 21%. Low mammography screening coverage has been reported in many LAC countries [40], [41]. In general, lower economic strata, no enrollment in social security and lower educational levels were associated with lower early detection practices [42].

A survey conducted in 2006 by the Latin American and Caribbean Society of Medical Oncology (SLACOM) in 12 countries with breast cancer specialists revealed that 62% of them reported a delay greater than three months between the suspicion of cancer and a mammogram or clinical exam in their country [43]. Delays between suspicion and diagnosis of breast cancer have been related to late stage diagnosis and consequently poorer survival [44]. Studies from Brazil [45] and Mexico [46] showed delays between presentation to a doctor and diagnosis of 6–7 months, and 4–5 months in Peru [47]. In a study from Paraguay included in the review, patients of different types of cancer took a median of nine months to seek medical attention, and it took a median of six months to diagnose their disease [48]. A study from Colombia showed a median time between first consultation and diagnosis of 90 days [49].

As for stage-specific survival, we could compile results from 34,282 patients from 12 countries, and the results varied greatly across studies. The absence of age and the start date of patient follow-up prevented us to pursue meaningful statistical analysis and meta-analysis. Rather descriptive information extracted from each study are provided in the Supporting Information (S7 Table). In addition, most studies reporting stage-specific survival were hospital-based consecutive case series, and our sample included very few population-based studies.

In developing countries, longer times between diagnosis and start of treatment remain a challenge [50] in addition to long time delays between case suspicion and diagnosis [45–47,49,51]. In the survey by SLACOM, treatment initiation delay was not reported as a problem in participant LAC countries, as most cancer specialists reported the majority of breast cancer patients starting treatment in less than three months from definitive diagnosis [43]. In Brazil, treatment delay was identified as an important issue and a law was passed in 2012 establishing a maximum of 60 days between histopathological confirmation and the start of treatment. The effect of this law was not assessed for breast cancer; however, for gynecological cancers only a small difference was reported between waiting times before and after the law was implemented [52]. Longer times between breast cancer surgery and adjuvant therapy have been reported to negatively affect survival [53]. One study from Brazil included in the review showed that each month of delay between surgery and the first adjuvant treatment increased the risk of death by 30% [54]. This is also a concern that needs to be addressed along with shortening the time periods between suspicion of breast cancer and diagnosis.

In this review, we used a broad search strategy and conducted in depth manual searches to gather the existing information about stage at diagnosis of more than 200,000 women with breast cancer in LAC. In spite of this, our analysis has a number of significant limitations. First, we were unable to find articles or reports from 27 countries. Second, for stage-specific survival estimates, we gathered information of more than 30,000 women, but even fewer countries were represented. Also, the studies did not provide the detailed classification of tumor and lymph node of their patients (eg., T1b, L1 etc), so we were unable to standardize the stage grouping considering the most recent TNM staging manual [55]. This would be important as patients then classified as early stage could have been reclassified as late stage, correcting the potential underestimation of the percentage of late stage diagnosis. As for stage-specific survival, it is known that stage migration affects survival, as patients with better prognosis migrate to a worst prognosis group, survival increases—the so-called Will Rogers phenomenon [56].

In addition, we were unable to compare survival estimates because it was not possible to adjust the results for age, and also because of the great heterogeneity in settings and patient case mixes in our study sample, that prevented comparability across studies. Fifth, very few population-based studies were included in our study sample. Lastly and most importantly, the great majority of the studies did not provide any information of ethnic distribution of their populations and of their socioeconomic statues and crucially of the kinds of health services (e.g., breast cancer screening) which were available to the patients included in our sample. LAC countries vary enormously by sociodemographic characteristics but even more so by the type and quality of their health care system and its financing.

LAC countries face the surge of chronic non-communicable diseases in the context of largely fragmented health systems and substantial socioeconomic inequalities [57]. Breast cancer is the leading cause of cancer death among women in the region and thus should receive considerable attention from local governments. In this review, we point to the large proportion of advanced disease diagnosis in the region. LAC countries should address concerns with early detection and diagnosis of breast cancer, and when financially sustainable implement appropriate screening and treatment programs.

## Supporting information

S1 Fig Publication bias analysis. Funnel chart.
(PDF)

S1 File Systematic review protocol. (PDF)

S2 File Search strategy. (PDF)

S3 File References of the included studies. (PDF)

S1 Table PRISMA 2009 Checklist. (PDF)

S2 Table Quality assessment of studies evaluating stage at diagnosis. (PDF)

S3 Table Quality assessment of studies evaluating survival probability. (PDF)

S4 Table Characteristics of the studies included for the outcome of stage at diagnosis. (PDF)

S5 Table Percentage of patients diagnosed at each stage. (PDF)

S6 Table Characteristics of the studies included for the outcome survival probability. (PDF)

S7 Table Survival results of the included studies. (PDF)
