# Supplementary material for: Stage at diagnosis and stage-specific survival of breast cancer in Latin America and the Caribbean: A systematic review and meta-analysis
Source: PLoS One. 2019 Oct 16;14(10):e0224012. doi: 10.1371/journal.pone.0224012 (PMC6799865; doi:10.1371/journal.pone.0224012)
Supplement: S1 Fig — Funnel chart. (PDF) [file pone.0224012.s001.pdf]

# S1 Fig. Publication Bias Analysis

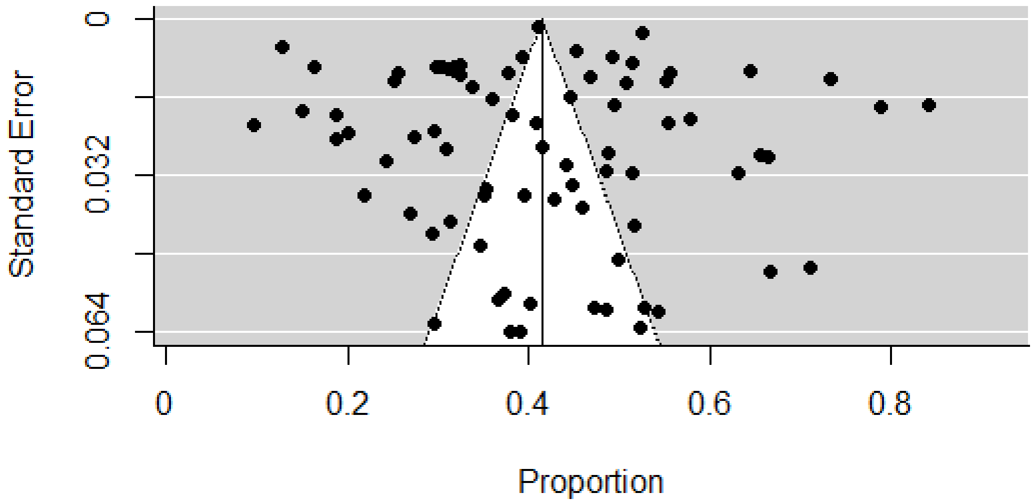


**S1 Fig. Funnel chart**

Regression Test for Funnel Plot Asymmetry

model: mixed-effects meta-regression model
predictor: standard error

test for funnel plot asymmetry: z = 0.9613, p = 0.3364
