# Supplementary material for: Stage at diagnosis and stage-specific survival of breast cancer in Latin America and the Caribbean: A systematic review and meta-analysis
Source: PLoS One. 2019 Oct 16;14(10):e0224012. doi: 10.1371/journal.pone.0224012 (PMC6799865; doi:10.1371/journal.pone.0224012)
Supplement: S1 File — (PDF) [file pone.0224012.s002.pdf]

S1 File: Systematic Review Protocol

Review title: Stage at presentation and stage-specific survival of female breast cancer in Latin America and the Caribbean: a systematic review of the literature

Review question:

What is the proportion of patients diagnosed with advanced stage breast cancer in Latin America and the Caribbean?

What is the survival probability by stage of female breast cancer in Latin America and the Caribbean?

Searches:

We will search the following electronic bibliographic databases: MEDLINE, EMBASE and LILACS (Latin American and Caribbean Health Sciences Literature). The search strategy will include the terms: "breast cancer" (and synonyms), and the names of Latin American and Caribbean countries (as defined by the United Nations), along with adjectives describing nationality (e.g., Argentina OR Argentinean).

Additional searches will be conducted on:

- the reference lists of included studies

- systematic reviews retrieved during the searches

- PAHO Virtual Health Library regional databases (Argentina, Belize, Bolivia, Brazil, Chile, Colombia, Costa Rica, Cuba, Ecuador, Guatemala, Guyana, Honduras, Mexico, Nicaragua, Panama, Paraguai, Peru, Puerto Rico, Dominican Republic, Trinidad and Tobago, Uruguay)

- Scientific Electronic Library Online (SciELO) regional databases (Argentina, Bolivia, Brazil, Chile, Colombia, Costa Rica, Cuba, Mexico, Peru, Uruguay and Venezuela)

- MedCarib (via PAHO Virtual Health Library)

Grey literature (abstracts, thesis and reports) retrieved by the additional searches will be considered for inclusion for countries for which we do not include any peer-reviewed articles.

The searches will be re-run prior to the final analysis and additional studies retrieved for inclusion.

Participants/population:

We will include studies evaluating women:

- With confirmed diagnosis of invasive breast cancer; AND

- Living in Latin American and the Caribbean.

We will not evaluate:

- Patients diagnosed with Phyllodes tumor of the breast;

- Patients born in Latin Americans and Caribbean living outside Latin America and the Caribbean;

- Pregnant and lactating women;

- Male patients*

*Considering that the incidence of male breast cancer is very low, studies that involve both sexes will not be excluded, even if results are not presented separately.

Types of study to be included:

Inclusion criteria:

- Observational studies (case series, cohort studies, cross-sectional studies, nested case-controlled studies, hospital based registries and population-based registries) reporting stage at diagnosis and/or stage-specific survival probability;

- Clinical trials reporting stage-specific survival of breast cancer;

- Studies published in any language (no language restrictions will be imposed).

Exclusion criteria:

- Studies not conducted in humans;

- Studies not conducted with Latin America and/or the Caribbean populations;

- Studies reporting only Hazard Ratios/Odds Ratios;

- Studies reporting aggregated stages including in situ stage (e.g., 0-IIa)

- Studies with a smaller population from the same location/registry of an included study (to avoid inclusion of repeated patients);

- Studies assessing patients with a specific disease stage (for stage at presentation);

- Multi-country studies that do not present results per country.

Main outcomes:

1) Proportion of patients diagnosed with advanced-stage breast cancer (stages III-IV).

2) Five-year stage-specific survival of patients diagnosed with invasive breast cancer.

Timing and effect measures

1) We will calculate the proportion of patients diagnosed with advanced stage breast cancer by subtracting the number of stage III-IV patients from the total number of staged patients. This value will be divided by the total number of staged patients and multiplied by 100 to obtain the percentage.

2) Five-year survival of patients with invasive breast cancer with T0 defined as the date of diagnosis and failure defined as death from all causes.

Additional outcome(s):

1) Proportion of patients diagnosed with late stage breast cancer (as considered by authors).

2) Stage-specific survival of patients diagnosed with invasive breast cancer in other time points.

Timing and effect measures

1) We will calculate the proportion of patients diagnosed with late stage advanced breast cancer, as considered by authors, by subtracting the number of late stage patients from the total number of staged patients. This value will be divided by the total number of staged patients and multiplied by 100 to obtain the percentage.

2) Survival probability of each stage or aggregated stage (e.g., I-II) at any time from T0 which may any given date (date of diagnosis, date of treatment start, date of hospitalization etc.). Failure may be death from all causes or cancer related death. Age-standardized relative survival at any time from T0 will also be collected.

Data extraction (selection and coding):

The titles and/or abstracts of articles retrieved will be screened to identify studies which potentially meet the inclusion criteria. The full texts of these articles will then be retrieved and comprehensively assessed for eligibility. All articles from the same study will be screened, even if they have not been retrieved by the search strategy, or were not considered to be potentially eligible after title/abstract screening. A final list of articles for inclusion will be determined after this process, and from these, data collection will be performed using a standardized, pre-piloted form.

Extracted information will include: study setting; study population; details of the intervention/exposure and control conditions (for clinical trials and comparative effectiveness studies); recruitment and study completion rates (for clinical trials); study methodology; stage at presentation; histopathologic results; survival probability (global and/or stage-specific); and information for assessment of the risk of bias and quality. Missing data will be requested from study authors.

Eligibility assessment and data collection will be performed in duplicate and independently by the review authors, with discrepancies being identified and resolved through discussion (with a third author, if necessary).

Risk of bias (quality) assessment:

Included studies will be evaluated according to the outcome of interest (stage at presentation or survival). Studies included for both outcomes will be evaluated for each outcome separately. Two reviewers will independently conduct the assessment using a standardized form that will capture three domains: minimizing selection bias; minimizing information bias; and assessment of other important variables.

The assessment of the study quality was inspired by the quality assessment tool designed by Eng *et al.* [1] and adapted by Jedy-Agba *et al.* 2016 [2]. For studies evaluating stage

at presentation the assessment is exactly the same from Jedy-Agba *et al.* [2] with exception of the addition of item 3.5.

The items for the quality assessment of studies evaluating survival were defined taking into account the sources of bias in longitudinal studies exemplified by Chubak *et al.* [3] and The Newcastle-Ottawa Scale (NOS) for assessing the quality of nonrandomized studies in meta-analyses [4].

The overall quality of each study will be expressed as the sum of its item-specific scores. The higher the score attained by a study, the higher the methodological quality, that is, the lower the risk that its findings may have been affected by bias.

Strategy for data synthesis:

We will analyze data with meta-analysis depicting (i) the proportion of patients diagnosed in advanced stages (as reported by authors) in each study and (ii) the five-year survival probability from diagnosis. Potential sources of heterogeneity will be evaluated with meta-regression. We will use de random-effect model. We will consider heterogeneity present when the P value of the Cochran's Q test is $< .1$ and I^2 statistic is $>50\%$. Potential publication bias was estimated with the Egger's test.

The meta-analysis of the proportion of patients diagnosed in advanced stages will be conducted using the R package Meta (metaprop function) [5,6]. To be included studies must directly report the proportion of patients diagnosed in advanced stages or give ways for this to be calculated.

The meta-analysis of survival will be conducted using the R package metafor (rma.mv function) [7]. To be included studies must provide the standard error or the number of

patients at risk and the number of events at five years. Each stage will be analyzed as a subgroup.

Analysis of subgroups or subsets:

The region of residence (Central America, Caribbean, and South America) will be considered for stage at presentation and survival assessments.

References

1. Eng A, McCormack V, dos-Santos-Silva I. Receptor-defined subtypes of breast cancer in indigenous populations in Africa: a systematic review and meta-analysis. *PLoS Med* 2014; **11**: e1001720.

2. Jedy-Agba E, McCormack V, Adebamowo C, Dos-Santos-Silva I. Stage at diagnosis of breast cancer in sub-Saharan Africa: a systematic review and meta-analysis. *Lancet Glob Health* 2016; **4**: e923–35.

3. Chubak J, Boudreau DM, Wirtz HS, McKnight B, Weiss NS. Threats to validity of nonrandomized studies of postdiagnosis exposures on cancer recurrence and survival. *J Natl Cancer Inst* 2013; **105**: 1456–62.

4. Wells GA, Shea B, O'Connell D, *et al.* The Newcastle-Ottawa Scale (NOS) for assessing the quality of nonrandomised studies in meta-analyses. The Ottawa Hospital - Research Institute. http://www.ohri.ca/programs/clinical_epidemiology/oxford.asp (accessed Nov 21, 2017).

5. Schwarzer G. meta: An R Package for Meta-Analysis. 2007.

6. Schwarzer G, Carpenter JR, Rücker G. Meta-analysis with R (Use-R!). *Switzerland: Springer International Publishing* 2015. https://svsl46kph01.storage.googleapis.com/MzMxOTIxNDE1Mg==01.pdf.

7. Viechtbauer W. Conducting meta-analyses in R with the metafor package. *J Stat Softw* 2010; **36**. http://brieger.esalq.usp.br/CRAN/web/packages/metafor/vignettes/metafor.pdf.
