# Supplementary material for: Stage at diagnosis and stage-specific survival of breast cancer in Latin America and the Caribbean: A systematic review and meta-analysis
Source: PLoS One. 2019 Oct 16;14(10):e0224012. doi: 10.1371/journal.pone.0224012 (PMC6799865; doi:10.1371/journal.pone.0224012)
Supplement: S2 File — (PDF) [file pone.0224012.s003.pdf]

S2 File: Search strategy

LILACS:

or "CANCER DE MAMA/EP") or "CANCER DA MAMA" or "CANCER DA MAMA/DI" or "CANCER DA MAMA/EP") or "CANCER DE SEIO" or "CANCER DE SEIO/DI" or "CANCER DE SEIO/EP" or "CANCER DE SENO" or "CANCER DE SENO/DI" or "CANCER DE SENO/EP") or "CANCER MAMARIO" or "CANCER MAMARIO/DI" or "CANCER MAMARIO/EP") or "NEOPLASIAS MAMARIAS") or "TUMORES DA MAMA" or "TUMORES DA MAMA/DI" or "TUMORES DA MAMA/EP") or "TUMORES DE MAMA" or "TUMORES DE MAMA/DI" or "TUMORES DE MAMA/EP") or "TUMOR DE SEIO" or "TUMOR DE SEIO/DI" or "TUMOR DE SEIO/EP" or "TUMOR DE SENO" or "TUMOR DE SENO/DI" or "TUMOR DE SENO/EP") or "TUMORES DE SEIO" or "TUMORES DE SEIO/DI" or "TUMORES DE SEIO/EP" or "TUMORES DE SENO" or "TUMORES DE SENO/DI" or "TUMORES DE SENO/EP") or "TUMORES MAMARIOS" or "TUMORES MAMARIOS/DI") or "CANCER OF BREAST" or "CANCER OF BREAST/DI" or "CANCER OF BREAST/EP") or "BREAST CANCER" or "BREAST CANCER/DI" or "BREAST CANCER/EP") or "BREAST TUMORS" or "BREAST TUMORS/DI" or "BREAST TUMORS/EP" or "BREAST-CANCER" [Palavras] and ("ANGUILLA") or "ANTIGUA AND BARBUDA") or "ANTIGUA AND BARBUDA/EP") or "ANTIGUA E BARBUDA") or "ANTIGUA E BARBUDA/EP") or "ANTIGUA Y BARBUDA") or "ANTIGUA Y BARBUDA/EP") or "BARBUDA") or "BARBUDA/EP") or "BAHAMAS") or "BAHAMAS/EP") or "British Virgin Islands") or "Islas Virgenes Britanicas") or "Ilhas Virgens Britanicas") or "cayman islands") or "ilhas cayman") or "Islas Caiman") or "CUBA") or "NETHERLANDS ANTILLES") or "ANTILLAS HOLANDESAS") or "ANTILHAS HOLANDESAS") or "BONAIRE") or "CURACAO") or "ST. EUSTATIUS") or "SANTO EUSTAQUIO") or "FEDERACAO AUTONOMA DOS PAISES BAIXOS") or "ANTILLAS NEDERLANDESAS") or "CURASAO") or "CURAZAO") or "SAN EUSTAQUIO") or "DOMINICA") or "DOMINICAN REPUBLIC") or "REPUBLICA DOMINICANA") or "GRENADA") or "HAITI") or "HAITI/EP") or "JAMAICA") or "JAMAICA/EP") or "MARTINIQUE") or "MARTINIQUE/EP") or "MARTINICA") or "MARTINICA/EP") or "WEST INDIES") or "WEST INDIES REGION") or "WEST INDIES REGION/EP") or "WEST INDIES/EP") or "INDIAS OCCIDENTALES") or "INDIAS OCCIDENTALES/EP") or "INDIAS OCIDENTAIS") or "INDIAS OCIDENTAIS/EP") or "ISLAS DEL CARIBE") or "ISLAS DEL CARIBE/EP") or "MONTSERRAT") or "MONTSERRAT/EP") or "ILHAS DO CARIBE") or "ILHAS DO CARIBE/EP") or "PUERTO RICO") or "PUERTO RICO/EP") or "PUERTO RICO") or "PUERTO RICO/EP") or "PORTO RICO") or "PORTO RICO/EP") or "saint barthelemy") or "San Bartolomé") or "São Bartolomeu") or "SAINT KITTS AND NEVIS") or "SAINT KITTS AND NEVIS/EP") or "SAN KITTS Y NEVIS") or "SAN KITTS Y NEVIS/EP") or "SAO CRISTOVAO E NEVIS") or "SAO CRISTOVAO E NEVIS/EP") or "SAINT LUCIA") or "SAINT LUCIA/EP") or "SANTA LUCIA") or "SANTA LUCIA/EP") or "saint martin") or "San Martin") or "SAINT VINCENT AND THE GRENADINES") or "SAINT VINCENT AND THE GRENADINES/EP") or "SAN VICENTE Y LAS GRENADINAS") or "SAN VICENTE Y LAS

GRENADINAS/EP") or "SAO VICENTE E GRANADINAS") or "SAO VICENTE E GRANADINAS/EP") or "GRENADINES") or "GRENADINES/EP") or "GRENADINAS") or "GRENADINAS/EP") or "GRANADINAS") or "GRANADINAS/EP") or "Sint maarten") or "sao martinho") or "TRINIDAD AND TOBAGO") or "TRINIDAD AND TOBAGO/EP") or "TRINIDAD E TOBAGO") or "TRINIDAD E TOBAGO/EP") or "TRINIDAD Y TOBAGO") or "TRINIDAD Y TOBAGO/EP") or "TRINIDADE") or "turks and caicos islands") or "Islas Turcas y Caicos") or "Ilhas Turks e Caicos") or "UNITED STATES VIRGIN ISLANDS") or "UNITED STATES VIRGIN ISLANDS/EP") or "VIRGIN ISLANDS OF THE UNITED STATES") or "VIRGIN ISLANDS OF THE UNITED STATES/EP") or "ISLAS VIRGENES DE LOS ESTADOS UNIDOS") or "ISLAS VIRGENES DE LOS ESTADOS UNIDOS/EP") or "ISLAS VIRGENES DE LOS ESTADOS UNIDOS") or "ISLAS VIRGENES DE LOS ESTADOS UNIDOS/EP") or "ILHAS VIRGENS AMERICANAS") or "ILHAS VIRGENS AMERICANAS/EP") or "BELIZE") or "BELIZE/EP") or "BRITISH HONDURAS") or "BRITISH HONDURAS/EP") or "BELICE") or "BELICE/EP") or "HONDURAS BRITANICAS") or "HONDURAS BRITANICAS/EP") or "COSTA RICA") or "COSTA RICA/EP") or "EL SALVADOR") or "EL SALVADOR/EP") or "GUATEMALA") or "GUATEMALA/EP") or "HONDURAS") or "HONDURAS/EP") or "MEXICO") or "MEXICO/EP") or "MEXICO/EPIDEMIOLOGY") or "NICARAGUA") or "NICARAGUA/EP") or "PANAMA") or "PANAMA/EP") or "ARGENTINA") or "ARGENTINA/EP") or "BOLIVIA") or "BOLIVIA/EP") or "bouvet island") or "Ilha bouvet") or "Isla bouvet") or "BRAZIL") or "BRAZIL-EPIDEMIOLOGY") or "BRAZIL/EP") or "BRASIL") or "BRASIL-EPIDEMIOLOGIA") or "BRASIL/EP") or "CHILE") or "CHILE/EP") or "COLOMBIA") or "COLOMBIA/EP") or "ECUADOR") or "ECUADOR/EP") or "EQUADOR") or "EQUADOR/EP") or "GALAPAGOS ISLANDS") or "GALAPAGOS ISLANDS/EP") or "ISLAS GALAPAGOS") or "ISLAS GALAPAGOS/EP") or "ILHAS GALAPAGOS") or "ILHAS GALAPAGOS/EP") or "FALKLANDS") or "Falkland Islands") or "MALVINAS") or "ILHAS MARIANAS") or "FRENCH GUIANA") or "FRENCH GUIANA/EP") or "GUYANA FRANCESA") or "GUYANA FRANCESA/EP") or "GUIANA FRANCESA") or "GUIANA FRANCESA/EP") or "GUYANA") or "GUYANA/EP") or "BRITISH GUIANA") or "BRITISH GUIANA/EP") or "GUYANA BRITANICA") or "GUYANA BRITANICA/EP") or "GUIANA") or "GUIANA BRITANICA") or "GUIANA BRITANICA/EP") or "PARAGUAY") or "PARAGUAY/EP") or "PARAGUAI") or "PARAGUAI/EP") or "PERU") or "PERU/EP") or "south georgia and the south sandwich islands") or "Georgia del sur y las islas Sandwich del sur") or "Geórgia do Sul e Sandwich do Sul") or "SURINAME") or "SURINAME/EP") or "DUTCH GUIANA") or "DUTCH GUIANA/EP") or "NETHERLANDS GUIANA") or "NETHERLANDS GUIANA/EP") or "GUYANA DE HOLANDA") or "GUYANA DE HOLANDA/EP") or "GUYANA HOLANDESA") or "GUYANA HOLANDESA/EP") or "SURINAM") or "SURINAM/EP") or "GUIANA HOLANDESA") or "GUIANA HOLANDESA/EP") or "URUGUAY") or "URUGUAY/EP") or "ORIENTAL REPUBLIC OF URUGUAY") or "REPUBLICA ORIENTAL DEL URUGUAY") or "REPUBLICA ORIENTAL DEL URUGUAY/EP") or "URUGUAI") or "URUGUAI/EP") or "REPUBLICA ORIENTAL DO URUGUAI") or "REPUBLICA ORIENTAL DO URUGUAI/EP") or "VENEZUELA") or "VENEZUELA/EP" [Palavras]

EMBASE:

(#1 'breast cancer'/exp OR 'advanced breast cancer' OR 'breast cancer recurrence' OR 'cancer, breast' OR 'mamma cancer' OR 'mammary cancer' OR 'mammary gland cancer' #2 'caribbean'/exp OR 'caribbean region' OR 'south and central america'/exp OR 'america, south and central' OR 'latin america' OR 'south america'/exp OR 'america, south' OR 'central america'/exp OR 'america, central' OR 'anguilla' OR 'antigua and barbuda' OR 'barbuda' OR 'aruba' OR 'bahamas' OR 'bahamian' OR 'barbados' OR 'bonaire, sint eustatius and saba' OR 'british virgin islands' OR 'cayman islands' OR 'cuba' OR 'cuban' OR 'curacao' OR 'dominica' OR 'dominican' OR 'dominican republic' OR 'grenada' OR 'grenadian' OR 'haiti' OR 'haitian' OR 'jamaica' OR 'jamaican' OR 'martinique' OR 'montserra' OR 'puerto rico' OR 'puerto rican' OR 'saint barthelemy' OR 'saint kitts and nevis' OR 'saint lucia' OR 'saint martin french part' OR 'saint vincent and the grenadines' OR 'vincentian' OR 'sint maarten dutch part' OR 'trinidad and tobago' OR 'trinbagonian' OR 'turks and caicos islands' OR 'united states virgin islands' OR 'belize' OR 'belizean' OR 'costa rica' OR 'costa rican' OR 'el salvador' OR 'salvadoran' OR 'guatemala' OR 'guatemalan' OR 'honduras' OR 'honduran' OR 'mexico' OR 'mexican' OR 'nicaragua' OR 'nicaraguan' OR 'panama' OR 'panamanian' OR 'argentinean' OR 'argentina' OR 'bolivia' OR 'bolivian' OR 'bouvet island' OR 'brazil' OR 'brazilian' OR 'chile' OR 'chilean' OR 'colombia' OR 'colombian' OR 'ecuador' OR 'ecuadorian' OR 'falkland islands' OR 'malvinas' OR 'french guiana' OR 'guyana' OR 'guyanese' OR 'paraguay' OR 'paraguayan' OR 'peru' OR 'peruvian' OR 'south georgia and the south sandwich islands' OR 'suriname' OR 'surinamensis' OR 'uruguay' OR 'uruguayan' OR 'venezuela bolivariana republic of' OR 'venezuela' OR 'venezuelan' #3 'animal'/exp OR 'animal population groups' OR 'animalia' OR 'animals' OR 'metazoa' OR 'metazoan' OR 'metazoans' OR 'metazoon' 1# AND #2 NOT #3

Medline (via Pubmed):

(((((breast cancer* or breast neoplasm* or breast carcinoma* or breast sarcoma* or breast tumor* or breast tumour* or breast malignanc*))) OR "Breast Neoplasms"[Mesh])) AND ((("Caribbean Region"[Mesh]) OR "Latin America"[Mesh]) OR (((caribbean) OR central america) OR south america) OR (((caribbean) OR central america) OR south america)) OR anguilla) OR ((antigua and barbuda))) OR barbuda) OR aruba) OR bahamas) OR bahamian) OR barbados) OR ((bonaire, sint eustatius and saba))) OR british virgin islands) OR cayman islands) OR cuba) OR cuban) OR curacao) OR dominica) OR dominican) OR dominican republic) OR grenada) OR grenadian) OR haiti) OR haitian) OR jamaica) OR jamaican) OR martinique) OR montserrat) OR puerto rico) OR puerto rican) OR saint barthelemy) OR ((saint kitts and nevis))) OR saint lucia) OR saint martin (french part)) OR ((saint vincent and the grenadines))) OR vincentian) OR sint maarten (dutch part)) OR ((trinidad and tobago))) OR trinbagonian) OR ((turks and caicos islands))) OR united states virgin islands) OR belize) OR belizean) OR costa rica) OR costa rican) OR el salvador) OR salvadoran) OR guatemala) OR guatemalan) OR honduras) OR honduran) OR mexico) OR mexican) OR nicaragua) OR nicaraguan) OR panama) OR panamanian) OR argentina) OR argentinean) OR argentina) OR bolivia) OR bolivian) OR bouvet island) OR brazil) OR brazilian) OR chile) OR chilean) OR colombia) OR colombian) OR ecuador) OR ecuadorian) OR falkland islands (malvinas)) OR french guiana) OR guyana) OR guyanese) OR paraguay) OR paraguayan) OR peru) OR peruvian) OR ((south georgia and the south sandwich islands))) OR suriname) OR surinamensis) OR uruguay) OR uruguayan) OR venezuela (bolivariana republic of)) OR venezuela) OR Venezuelan[all]))))))

Cochrane Central:

#1MeSH descriptor: [Breast Neoplasms] explode all trees #2Breast Neoplasms (Word variations have been searched) #3breast neoplasm (Word variations have been searched) #4breast carcinoma (Word variations have been searched) #5breast sarcoma (Word variations have been searched) #6breast tumor (Word variations have been searched) #7breast malignanc (Word variations have been searched) #8 {or#1-#7} #9MeSH descriptor: [Caribbean Region] explode all trees #10Caribbean Region)Word variations have been searched) #11 MeSH descriptor: [Latin America] explode all trees #12 Latin America (Word variations have been searched) #13 caribbean (Word variations have been searched) #14central america (Word variations have been searched) #15south america (Word variations have been searched) #16caribbean (Word variations have been searched) #17central america (Word variations have been searched) #18south america (Word variations have been searched) #19anguilla (Word variations have been searched) #20 antigua and barbuda (Word variations have been searched) #21barbuda (Word variations have been searched) #22aruba (Word variations have been searched) #23bahamas (Word variations have been searched) #24bahamian (Word variations have been searched) #25barbados (Word variations have been searched) #26 bonaire, sint eustatius and saba (Word variations have been searched) #27british virgin islands (Word variations have been searched) #28cayman islands (Word variations have been searched) #29cuba (Word variations have been searched) #30 cuban (Word variations have been searched) #31curacao (Word variations have been searched) #32dominica (Word variations have been searched) #33dominican (Word variations have been searched) #34dominican republic (Word variations have been searched) #35 grenada (Word variations have been searched) #36 grenadian (Word variations have been searched) #37 haitian (Word variations have been searched) #38haiti (Word variations have been searched) #39jamaica (Word variations have been searched) #40 jamaican (Word variations have been searched) #41 martinique (Word variations have been searched) #42 montserrat (Word variations have been searched) #43puerto rico (Word variations have been searched) #44puerto rican (Word variations have been searched) #45puerto rican (Word variations have been searched) #46saint kitts and nevis (Word variations have been searched) #47saint lucia (Word variations have been searched) #48saint martin (Word variations have been searched) #49saint vincent and the grenadines (Word variations have been searched) #50 vincentian (Word variations have been searched) #51sint maarten (Word variations have been searched) #52trinidad and tobago (Word variations have been searched) #53trinbagonian (Word variations have been searched) #54turks and caicos islands (Word variations have been searched) #55united states virgin islands (Word variations have been searched) #56belize (Word variations have been searched) #57belizean (Word variations have been searched) #58 costa rica (Word variations have been searched) #59costa rican (Word variations have been searched) #60el salvador (Word variations have been searched) #61salvadoran (Word variations have been searched) #62guatemala (Word variations have been searched) #63guatemalan (Word variations have been searched) #64honduras (Word variations have been searched) #65 honduran (Word variations have been searched) #66 mexico (Word variations have been searched) #67mexican (Word variations have been searched) #68nicaragua (Word variations have been searched) #69nicaraguan (Word variations have been searched) #70 panama (Word variations have been searched) #71panamanian (Word variations have been searched) #72argentina (Word variations have been searched) #73argentinean (Word variations have been searched) #74bolivia (Word variations have been searched) #75 bolivian (Word variations have been searched) #76bouvet island (Word variations have been searched) #77brazil (Word variations have been searched) #78brazilian (Word

variations have been searched) #79chile (Word variations have been searched) #80 chilean (Word variations have been searched) #81colombia (Word variations have been searched) #82colombian (Word variations have been searched) #83ecuador (Word variations have been searched) #84ecuadorian (Word variations have been searched) #85falkland islands (malvinas) (Word variations have been searched) #86french guiana (Word variations have been searched) #87guyana (Word variations have been searched) #88 guyanese (Word variations have been searched) #89 paraguay (Word variations have been searched) #90paraguayan (Word variations have been searched) #91 peru (Word variations have been searched) #92peruvian (Word variations have been searched) #93south georgia and the south sandwich islands (Word variations have been searched) #94 surinamensis (Word variations have been searched) #95 uruguay (Word variations have been searched) #96uruguayan (Word variations have been searched) #97venezuela (bolivariana republic of) (Word variations have been searched) #98venezuela (Word variations have been searched) #99Venezuelan (Word variations have been searched) #100{or #9-#99} #101 #8 and #100
