# Supplementary material for: Stage at diagnosis and stage-specific survival of breast cancer in Latin America and the Caribbean: A systematic review and meta-analysis
Source: PLoS One. 2019 Oct 16;14(10):e0224012. doi: 10.1371/journal.pone.0224012 (PMC6799865; doi:10.1371/journal.pone.0224012)
Supplement: S3 File — (PDF) [file pone.0224012.s004.pdf]

S3 File: References of the included studies

In the main text, figures and tables the included studies as referred as: Country Code[1]-Name of first author(s) (date of publication). Below are the complete references of the studies organized by region (Caribbean, Central America, and South America)

Caribbean

Bahamas

BHS-Mungrue (2016)
Mungrue K, Chase H, Gordon J, et al. Breast Cancer in the Bahamas in 2009–2011. *Breast Cancer* 2016; **10**: BCBCR.S32792.

Barbados

BRB-Nemesure (2009)
Nemesure B, Wu S-Y, Hambleton IR, Leske MC, Hennis AJ, Barbados National Cancer Study Group. Risk factors for breast cancer in a black population--the Barbados National Cancer Study. *Int J Cancer* 2009; **124**: 174–9.

Cuba

CUB-Mora-Díaz (2004)
Mora-Díaz I, Sánchez-Redonet E. Estado actual de las pacientes con cáncer de mama en estadio I y II. *Revista Cubana de Obstetricia y Ginecología* 2004; **30**: 1–6.

CUB-Moreno de Miguel (1998)
Moreno de Miguel LF, Pérez-Braojo I, Sánchez-Varela I, Rodríguez-Díaz R. Cirugía conservadora+ radioterapia en el cáncer temprano de mama. *Rev Cubana de Oncol* 1998; **14**: 143–8.

CUB-Ricardo-Ramírez (2013)
Ricardo-Ramírez JM, Mustelier-Santana LR, Pérez-Acosta J, Ferrer-Aguirre M, Romero-García LI. Supervivencia y seguimiento clínico de mujeres mastectomizadas durante una década. *MEDISAN* 2013; **17**: 4073–80.

CUB-Viera-Hernándé (2011)
Viera-Hernández RV, Amaro-Areas E, Barro-Blanco A, Rodríguez-Hernández A, Perez-Pozo M. Caracterización del cáncer de mama. Isla de la Juventud. 2000-2010. *Revista de Medicina Isla de la Juventud* 2011; **12**: 74–87.

CUB-Ruiz-Lorente (2011)
Ruiz-Lorente R, Hernández-Rubio MC, Durán-Hernández D, Lien-Tamayo TG. Experiencia en mujeres con cáncer de mama durante un trienio. *Rev Cubana Obstet Ginecol* 2011; **37**: 349–58.

---

[1] United Nations Statistics Division. UNSD — Methodology. https://unstats.un.org/unsd/methodology/m49/ (accessed Nov 28, 2018).

CUB-Gonzáles-Longoria Boada and Lemes-Báez (2011)
Gonzáles-Longoria Boada LB, Lemes-Báez JJ. Supervivencia del cáncer de mama. *Rev Arch Bibl Mus* 2011; **15**: 983–92.

CUB-Garrote (2011)
Garrote LF, Alvarez YG, Babie PT, Yi MG, Alvarez MG, Cicili ML. Cancer survival in Cuba, 1994-1995. *IARC Sci Publ* 2011; **10**: 89–95.

CUB-Fernandez-Garrote (1998)
Fernandez-Garrote L, Graupera-Boschmonar M, Galan-Alvarez Y, Lezcano-Cicilli M, Martin-Garcia A, Camacho-Rodriguez R. Cancer survival in Cuba. *IARC Sci Publ* 1998; **8**: 51–9.

CUB-Milián-Mosquera (2015)
Milián-Mosquera EN, Rodríguez-Feliz T, Justo-Celorrio MV, Batista-Serrano R, Algarin-Mariño JC. Características de pacientes con cáncer de mama diagnosticado en el año 2013 en el municipio de Las Tunas. *Revista Electrónica Dr Zoilo E Marinello Vidaurreta* 2015; **40**: 1-10 http://www.revzoilomarinello.sld.cu/index.php/zmv/article/view/37 (accessed April 6, 2018).

CUB-Gómez-Delgado (2017)
Gómez-Delgado I, Estévez-Cobo L, Estévez-Gómez L. Características clínico-epidemiológicas de pacientes con cáncer de mama. Hospital Universitario Dr. Celestino Hernández Robau, 2010-2014. *Medicentro Electrónica* 2017; **21**: 57–60.

Haiti

HTI-DeGennaro (2018)
DeGennaro V, Jiwani F, Patberg E, et al. Epidemiological, Clinical, and Histopathological Features of Breast Cancer in Haiti. *Journal of Global Oncology* 2018; **4**: 1–9.

Jamaica

JAM-Alfred (2012)
Alfred R, Chin SN, Williams E, Walters C, Barton EN, Shah D. The prevalence and significance of oestrogen receptor (ER) positivity in breast cancer at the University Hospital of the West Indies, Jamaica. *West Indian Med J* 2012; **61**: 795–801.

Puerto Rico

PRI-Ortiz (2010)
Ortiz AP, Frías O, González-Keelan C, et al. Clinicopathological factors associated to HER-2 status in a hospital-based sample of breast cancer patients in Puerto Rico. *P R Health Sci J* 2010; **29**: 265–71.

Trinidad and Tobago

TTO-Warner (2015)
Warner WA, Morrison RL, Lee TY, et al. Associations among ancestry, geography and breast cancer incidence, mortality, and survival in Trinidad and Tobago. *Cancer Med* 2015; **4**: 1742–53.

TTO-Raju and Naraynsingh (1989)
Raju GC, Naraynsingh V. Breast cancer in West Indian women in Trinidad. *Trop Geogr Med* 1989; **41**: 257–60.

Central America

Costa Rica

CRI-Quirós-Alpízar (2017)
Quirós-Alpízar JL, Espinoza-Morales K. Supervivencia a 5 años de pacientes con cáncer de mama triple negativo. *Medicina Legal de Costa Rica* 2017. **34**: 1–14. http://www.scielo.sa.cr/scielo.php?script=sci_arttext&pid=S1409-00152017000100059.

CRI-Ortiz-Barboza (2011)
Ortiz-Barboza A, Gomez L, Cubero C, Bonilla G, Mena H. Cancer survival in Costa Rica, 1995-2000. *IARC Sci Publ 2011*; **9**: 85–8.

Honduras

NHD-Muñoz (2011)
Muñoz FD, Cálix ES, Santos R. Caracterización Epidemiológica de Pacientes con Cáncer de Mama, Admitidas en el Centro de Cáncer ‘Emma Romero De Callejas’ 1999 a 2009. *Revista de la Facultad de Ciencias Médicas* 2011; **8**: 32–44.

Mexico

MEX-Pérez-Michel (2009)
Pérez-Michel L, González-Lizarraga M, Ornelas-Aguirre JM. Recurrencia de cáncer de mama en mujeres del Noroeste de México. *Cirugía y Cirujanos* 2009; **77**: 179–85.

MEX-Flores-Luna (2008)
Flores-Luna L, Salazar-Martínez E, Duarte-Torres RM, Torres-Mejía G, Alonso-Ruiz P, Lazcano-Ponce E. Factores pronósticos relacionados con la supervivencia del cáncer de mama. *Salud Pública Méx* 2008; **50**: 119–25.

MEX-Álvarez-Bañuelos (2016)
Álvarez-Bañuelos MT, Rosado-Alcocer LM, Morales-Romero J, Román-Álvarez LS, García REG-, Carvajal-Moreno M. Prognostic Factors Associated with Survival in Women with Breast Cancer from Veracruz, Mexico. *J Cancer Sci Ther* 2016; **8**. DOI:10.4172/1948-5956.1000398.

MEX-Arce-Salinas (2012)
Arce-Salinas C, Lara-Medina FU, Alvarado-Miranda A, et al. Evaluación del tratamiento del cáncer de mama en una institución del tercer nivel con el Seguro Popular, México. *Revista de Investigación Clínica* 2012; **64**: 9–16.

MEX-Ángeles-Llerenas (2016)
Ángeles-Llerenas A, Torres-Mejía G, Lazcano-Ponce E, et al. Effect of care-delivery delay on the survival of Mexican women with breast cancer. *Salud Pública Mex* 2016; **58**: 237–50.

MEX-Lara-Medina (2011)
Lara-Medina F, Pérez-Sánchez V, Saavedra-Pérez D, et al. Triple-negative breast cancer in Hispanic patients: high prevalence, poor prognosis, and association with menopausal status, body mass index, and parity. *Cancer* 2011; **117**: 3658–69.

MEX- Reynoso-Noverón (2017)
Reynoso-Noverón N, Villarreal-Garza C, Soto-Perez-de-Celis E, et al. Clinical and Epidemiological Profile of Breast Cancer in Mexico: Results of the Seguro Popular. *J Glob Oncol* 2017; **3**: 757–64.

MEX- di Filippo-Echeverri (2004)
di Filippo-Echeverri B, Miranda-Hernández H, Luján-Castilla P, Serrano-Migallón J, Ávila-Medrano L, Pacheco-Álvarez I. Manejo conservador del cáncer mamario. Experiencia en el servicio de Oncología del Hospital General de México. *Gaceta Mexicana de Oncología* 2004; **3**: 59–63.

MEX-Maffuz-Aziz (2016)
Maffuz-Aziz A, Labastida-Almendaro S, Sherwell-Cabello S, et al. Supervivencia de pacientes con cáncer de mama. Análisis por factores pronóstico, clínicos y patológicos. *Ginecol Obstet Mex* 2016; **84**: 498–506.

MEX-Ortega-Cervantes (2013)
Ortega-Cervantes L, Rojas-García AE, Robledo-Marenco M de L, et al. Morbidity of breast cancer and cervico-uterine cancer in women from the occidental region of Mexico. *Rev Invest Clin* 2013; **65**: 221–7.

MEX-Ramírez-Torres (2016)
Ramírez-Torres N, Pérez-Puentes A, Rivas-Ruiz R, Talavera JO, Astudillo-de la Vega H. Impacto pronóstico de la respuesta patológica completa y del estado ganglionar en pacientes con cáncer de mama avanzado tratadas con dosis alta de epirrubicina neoadyuvante. *Gaceta Mexicana de Oncología* 2016; **15**: 128–37.

MEX-Leon-Rodriguez (2017)
Leon-Rodriguez E, Molina-Calzada C, Rivera-Franco MM, Campos-Castro A. Breast self-exam and patient interval associate with advanced breast cancer and treatment delay in Mexican women. *Clin Transl Oncol* 2017; **19**: 1276–82.

MEX-Medina-Franco (2017)
Medina-Franco H, Gaona-Luviano P. Disparities in Breast Cancer Characteristics in Mexico. *GAMO* 2017; **16**. DOI:10.24875/j.gamo.17000017.

South America

Argentina

ARG-Bianco (1985)
Bianco M, Heller L, Meiss R. Estudio epidemiologico colaborativo de cancer de mama en la argentina. *Bol A N de Medicina* 1985; **63**: 485–94.

Arg-Juarez (2009)
Juarez AM. Edad y estadio de las mujeres con cáncer de mama. Hospitales públicos. Córdoba 1998/2003. *Revista de Salud Pública* 2009; **13**: 33–42.

ARG-Iturbe (2011)
Iturbe J, Zwenger A, Leone JP, et al. Treatment of early breast cancer, a long-term follow-up study: the GOCS experience. *Breast J* 2011; **17**: 630–7.

ARG-Arce (2013)
Arce C, Luque R, Ruiz Díaz L, et al. Experiencia sobre mil cánceres de mama en posadas, misiones. *Revista Argentina de Mastología* 2013; **32**: 148–59.

ARG-Berra (2016)
Berra MG, Morcos PS, Sarrouf MC, Galleano J, Sarrouf J. Influencia del perfil inmunohistoquímico sobre la sobrevida libre de enfermedad y la sobrevida global en Cáncer de Mama Localmente Avanzado. *Revista Argentina de Mastología* 2016; **36**: 75–89.

ARG-Elizalde (2013)
Elizalde R, Bustos J, Perrier GM, et al. Caracteristicas Epidemiologicas del Cancer de Mama en el Area Metropolitana de Buenos Aires y La Plata: Estudio de una serie de 4.041 casos del Registro de Cáncer de Mama (RCM). *Revista Latinoamericana de Mastologia* 2013; **7**. http://www.flamastologia.org/rlamastologia/index.php/journal/article/view/74.

ARG-Grippo (2015)
Grippo NM, Raineri E, Yapur R, Romero P, López Raffo MM. Análisis de las variables clinicopatológicas e inmunohistoquímicas del cáncer de mama. *Revista Argentina de Mastología* 2015; **34**: 14–26.

ARG-Meiss (2016)
Meiss RP, Chuit. R. Caracterización del Cáncer de Mama en mujeres de la República Argentina. *Revista Argentina de Mastología* 2016; **36**: 65–91.

ARG-Palazzo (2016)
Palazzo A, Perinetti A, Vacchino M. Estadio clínico del cáncer de mama y nivel socioeconómico en el partido de General Pueyrredón, Argentina, 2013. *Revista Argentina de Salud Pública* 2016; **7**: 16–20.

Brazil

BRA-Antunes (2015)
Antunes Y, Bugano DDG, Giglio A, Kaliks RA, Karnakis T, Pontes LB. Características clínicas e de sobrevida global em pacientes oncológicos idosos num centro oncológico terciário. *Einstein* 2015; **13**: 487–91.

BRA-Medeiros (2015)
Medeiros GC, Bergmann A, Aguiar SS de, Thuler LCS. Análise dos determinantes que influenciam o tempo para o início do tratamento de mulheres com câncer de mama no Brasil. *Cad Saúde Pública* 2015; **31**: 1269–82.

BRA-Stival (2012)
Stival RA, de Almeida Martins LR. Impacto do fenótipo triplo-negativo no prognóstico de pacientes com câncer de mama de uma unidade de referência no Brasil central. *Rev Bras Mastologia* 2012; **22**: 6–12.

BRA-Ayala (2012)
Ayala AML. Sobrevida de mulheres com câncer de mama, de uma cidade no sul do Brasil. *Rev Bras Enferm* 2012; **65**. http://www.redalyc.org/html/2670/267024790003/.

BRA-Guerra (2009)
Guerra MR, Mendonça GA e. S, Bustamante-Teixeira MT, Cintra JRD, Carvalho LM de, Magalhães LMPV de. Sobrevida de cinco anos e fatores prognósticos em coorte de pacientes com câncer de mama assistidas em Juiz de Fora, Minas Gerais, Brasil. *Cad Saúde Pública* 2009; **25**: 2455–66.

BRA-Schneider (2009)
Schneider IJC, d'Orsi E. Sobrevida em cinco anos e fatores prognósticos em mulheres com câncer de mama em Santa Catarina, Brasil. *Cad Saúde Pública* 2009; **25**: 1285–96.

BRA-Moraes (2006)
Moraes AB de, Zanini RR, Turchiello MS, Riboldi J, Medeiros LR de. Estudo da sobrevida de pacientes com câncer de mama atendidas no hospital da Universidade Federal de Santa Maria, Rio Grande do Sul, Brasil. *Cad Saúde Pública* 2006; **22**: 2219–28.

BRA-Thuler and Mendonça (2005)
Thuler LCS, Mendonça GA. Estadiamento inicial dos casos de câncer de mama e colo do útero em mulheres brasileiras. *Rev Bras Ginecol Obstet* 2005; **27**: 656–60.

BRA-Vazquez (2016)
Vazquez F de L, Silva TB, Vieira RA da C, et al. Retrospective analysis of breast cancer prognosis among young and older women in a Brazilian cohort of 738 patients, 1985-2002. *Oncol Lett* 2016; **12**: 4911–24.

BRA-Fayer (2016)
Fayer VA, Guerra MR, Cintra JRD, Bustamante-Teixeira MT. Ten-year survival and prognostic factors for breast cancer in the southeast region of Brazil. *Rev Bras Epidemiol* 2016; **19**: 766–78.

BRA-Carrara (2017)
Carrara GFA, Scapulatempo-Neto C, Abrahão-Machado LF, et al. Breast-conserving surgery in locally advanced breast cancer submitted to neoadjuvant chemotherapy. Safety and effectiveness based on ipsilateral breast tumor recurrence and long-term follow-up. *Clinics* 2017; **72**: 134–42.

Chile

CHL-Peralta (1995)
Peralta OM, Alfonso JM, Rencoret CV, Del Castillo CS, Solé JB, Campodónico IG. Cancer de Mama. Resultados del Programa de Pesquisa y Tratamiento del Servicio de Salud Central. *Rev Chil Obstet Ginecol* 1995; **60**: 417–27.

CHL-Prieto (2011)
Prieto MM. Epidemiología del cáncer de mama en chile. *Revista Médica Clínica Las Condes* 2011; **22**: 428–35.

CHL-Jurgensen (2009)
Jürgensen C M, Chacón C R, Baeza R C, Riveros P R. Cánceres de mama multifocales-multicéntricos: ¿Son realmente de peor pronóstico? *Rev Chil Cir* 2009; **61**. DOI:10.4067/S0718-40262009000200004.

CHL-Acevedo (2006)
Acevedo B, Carlos J, Rossat A, et al. Cáncer de mama: experiencia del Centro Integral de la Mama de Clínica Las Condes 1996-2005. *Rev Méd Clín Condes* 2006; **17**: 248–55.

Colombia

COL-González-Mariño (2005)
González-Mariño MA. Cáncer mamario Registro de Cáncer Clínica San Pedro Claver, 2003. *Revista Colombiana de Cirugía* 2005; **20**. http://www.redalyc.org/html/3555/355534449003/.

COL-Martínez (2012)
Martínez SP, Segura AR, Arias SA, Mateus G. Caracterización de los tiempos de atención y de mujeres con cáncer de mama que asistieron a un hospital de tercer nivel, 2005-2009. *Facultad Nacional de Salud Pública* 2012; **30**: 183–91.

COL-Pardo (2015)
Pardo C, de Vries E, Duarte JM, Piñeros M. Cáncer en la Unidad de Cáncer del Hospital Departamental de Villavicencio, Colombia, 2006-2008. *Revista Colombiana de Cancerología* 2015; **19**: 125–32.

COL-Piñeros (2008)
Piñeros M, Sánchez R, Cendales R, et al. Características sociodemográficas, clínicas y de la atención de mujeres con cáncer de mama en Bogotá. *Revista Colombiana de Cancerología* 2008; **12**: 181–90.

COL-Robledo-Abad (2005)
Robledo-Abad JF, Caicedo-Mallarino JJ, DeAntonio-Suárez R. Análisis de sobrevida en una cohorte de 1328 pacientes con carcinoma de seno. *Revista Colombiana de Cirugía* 2005; **20**: 4–20.

COL-Pardo (2003)

Pardo C, Murillo R, Piñeros M, Castro MÁ. Casos nuevos de cáncer en el Instituto Nacional de Cancerología, Colombia, 2002. *Revista Colombiana de Cancerología* 2003; **7**: 4–19.

COL-Ospino (2010)
Ospino R, Cendales R, Cifuentes J, Sánchez Z, Galvis J, Bobadilla I. Supervivencia en pacientes con cáncer de mama localmente avanzado tratadas con radioterapia posterior a mastectomía en el Instituto Nacional de Cancerología. *Revista Colombiana de Cancerología* 2010; **14**: 210–24.

COL-Ospino (2011)
Ospino R, Cendales R, Sánchez Z, Bobadilla I, Galvis J, Cifuentes J. Supervivencia en pacientes con cáncer de mama temprano tratadas con cirugía conservadora asociada a radioterapia en el Instituto Nacional de Cancerología. *Revista Colombiana de Cancerología* 2011; **15**: 75–84.

COL-Angarita (2010)
Angarita FA, Acuña SA, Torregrosa L, Tawil M, Ruiz ÁJ. Presentación inicial de las pacientes con diagnóstico de cáncer de seno en el Centro Javeriano de Oncología, Hospital Universitario San Ignacio. *Revista Colombiana de Cirugía* 2010; **25**: 19–26.

COL-González-Mariño (2006)
González-Mariño MA. Cáncer de seno en la Clínica San Pedro Claver de Bogotá, 2004. *Rev Salud Pública* 2006; **8**: 163–9.

Col-Zuluaga-Liberato (2016)
Zuluaga-Liberato A, Zuluaga-Cristancho A. Estadio de la enfermedad, receptores hormonales y sobreexpresión de HER2: factores pronósticos en cáncer de seno para una cohorte de Bogotá (2005-2013). *Revista Colombiana de Hematología y Oncología* 2016; **3**: 17–23.

COL-Lenis and Esparza (1998)
Lenis N, Esparza CA. Cáncer de mama: Diagnóstico, tratamiento y seguimiento. Hospital de Caldas. *Rev Col Cirurgia* 1998; **13**: 251–8.

COL-Ramírez-Martínez (2015)
Ramírez-Martínez C, Clavijo-Rodríguez J, Estrada-Restrepo JDJ, Restrepo-Ramírez CA. Description of clinic, anatomopathologic and treatment characteristics of pacients with breast cancer a senology unit in Medellin, Colombia; 2006-2013. *CES Medicina 2015*; **29**: 181–90.

COL-García (2012)
García Ó, Ossa CA, Beltrán MI, Cano M, Villamizar L, Arias AM. Descripción de una cohorte de pacientes con cáncer de mama triple-negativo subtipo basal-like, atendidas en el Instituto Nacional de Cancerología y en el Hospital de San José durante el periodo 2006-2008. *Revista Colombiana de Cancerología* 2012; **16**: 91–9.

Ecuador

ECU-Cueva and Yépez (2014)

Cueva P, Yépez J. Cancer Epidemiology in Quito 2006-2010. Sociedad de Lucha Contra el Cáncer - Solca Quito - *Registro Nacional De Tumores* 2014; **15**: 8–241.

ECU-Cueva and Yépez (2009)
Cueva P, Yépez J. Epidemiología del Cáncer en Quito 2003-2005. Sociedad de Lucha Contra el Cáncer - Solca Quito - Registro Nacional De Tumores 2009. http://www.solcaquito.org.ec/index.php/publicaciones/epidemiologia/cancer-en-quito-2003-2005 (accessed Feb 17, 2018).

ECU-Martínez (2015)
Martínez F, Abril L, Pérez L. Sexto Informe Registro de Tumores Cuenca 2005-2009. Instituto del Cáncer SOLCA Cuenca Epidemiología del Cáncer en el Cantón Cuenca 2015; 1: 1–400.

French Guiana

GUF-Roué (2016)
Roué T, Labbé S, Belliardo S, Plenet J, Douine M, Nacher M. Predictive Factors of the Survival of Women With Invasive Breast Cancer in French Guiana: The Burden of Health Inequalities. *Clin Breast Cancer* 2016; **16**: e113–8.

Guyana

GUY-Taioli (2010)
Taioli E, Attong-Rogers A, Layne P, Roach V, Ragin C. Breast cancer survival in women of African descent living in the US and in the Caribbean: effect of place of birth. *Breast Cancer Res Treat* 2010; **122**: 515–20.

Paraguay

PRY-Yoffe de Quiroz (2005)
Yoffe de Quiroz I. Retardo en el diagnóstico de los pacientes con cáncer. *Anales de la Facultad de Ciencias Médicas* 2005. **38**: 22–28 http://scielo.iics.una.py/scielo.php?pid=S1816-89492005000100003&script=sci_arttext.

Peru

PER-Días (1999)
Díaz J, Salgado L, Roeder R. Sobrevida con mastectomia radical en cancer de mama invasor. *Revista Peruana de Ginecología y Obstetricia* 1999; **45**: 97–1105.

PER-Díaz-Vélez (2013)
Díaz-Vélez C. Informe del registro Hospitalario de Cancer 2007-2012. Red Asistencial Lambayeque, 2013.

PER- Larrea-Fernandez (2016)
Larrea-Fernandez L. Características clínico patológicas del cáncer de mama en mujeres menores de cincuenta años, Hospital Nacional Guillermo Almenara Irigoyen, 2009 – 2010. *Revista Medica Carrionica* 2016; **3**.

http://cuerpomedico.hdosdemayo.gob.pe/index.php/revistamedicacarrionica/article/view/31 (accessed April 7, 2018).

PER-Gutiérrez and Alarcón (2008)
Gutiérrez C, Alarcón E. Nivel de pobreza asociado al estadio de gravedad del cáncer ginecológico. In: Anales de la Facultad de Medicina. UNMSM. Facultad de Medicina, 2008: 239–43.

PER-Infanzón (2000)
Infanzón M. Cáncer de mama en pacientes mayores de 70 años. *Ginecol e Obstet* 2000; **46**: 65–9.

Suriname

SUR-van Leeuwaarde (2011)
van Leeuwaarde RS, Vrede MA, Henar F, et al. A nationwide analysis of incidence and outcome of breast cancer in the country of Surinam, during 1994-2003. *Breast Cancer Res Treat* 2011; **128**: 873–81.

Uruguay

URY-Camejo (2015)
Camejo N, Castillo C, Richter L, et al. Evaluación de la calidad de la asistencia en la Unidad Docente Asistencial de Mastología del Hospital de Clínicas. *Revista Médica del Uruguay* 2015; **31**: 165–71.

URY-Vázquez (2005)
Vázquez T, Krygier G, Barrios E, et al. Análisis de sobrevida de una población con cáncer de mama y su relación con factores pronósticos: estudio de 1.311 pacientes seguidas durante 230 meses. *Revista Médica del Uruguay* 2005; **21**: 107–21.

URY-Malvasio (2012)
Malvasio S, Schiavone A, Camejo N. Características clínico-patológicas y evolución del cáncer de mama en mujeres uruguayas jóvenes. *Rev Méd Urug* 2017; **33**: 94-101.

Venezuela

VEN-Hung (2012)
Hung CY, López L, Lizardo A, et al. Expresión de KI-67 Como Factor Pronóstico en las Clases Moleculares de Carcinoma de Mama. *Revista Venezolana de Oncología* 2012; **24**: 107-24.

VEN-Godoy (2000)
Godoy AJB, Betancourt L, Taronna I, Martirené E, Higuerey J. Evaluación del Carcinoma de Mama Estadio III en el Instituto de Oncología 'Luis Razetti'. *Revista Venezolana de Oncología* 2000; **12**. http://www.oncologia.org.ve/site/upload/revista/pdf/Godoy.pdf.

VEN-Pachedo-Soler (2000)
Pacheco-Soler C, Barrios G, Tejada A, et al. Tratamiento del cáncer de mama en pacientes ancianas. *Revista Venezolana de Oncología* 2000; **12**: 56–66.

VEN-Ravelo-Celis (2007)
Ravelo-Celis JA, Ravelo-Pagés R. Tratamiento preservador del cáncer de la mama. Experiencia personal en 15 años. *Colección Razetti* 2007; **3**: 463–516.

VEN-Acosta-Marín (2011)
Acosta-Marín V, Acosta F. V, Marín E, et al. ¿Es carcinoma lobulillar infiltrante igual a carcinoma ductal infiltrante? Seguimiento a largo plazo. *Revista Venezolana de Oncología* 2011; **23**: 56–65.

VEN-Vera (2002)
Vera A, Urdaneta N, Gutiérrez E, et al. Veinte años de experiencia en el tratamiento del cáncer mamario precoz: con preservación del seno: análisis restrospectivo de 569 casos. *Revista Venezolana de Oncología* 2002; **14**: 66–73.

VEN-Ferri (2012)
Ferri N, Contreras AC, Payares E, et al. Cirugía del carcinoma mamario revisión de 20 años. *Revista Venezolana de Oncología* 2012; **24**: 132–42.

VEN-Rebolledo (2012)
Rebolledo VE, Ferri N, Reigosa A, Caleiras E, Fernández Y. Perfil inmunohistoquímico y la caracterización molecular del carcinoma de mama en una población venezolana. *Revista Venezolana de Oncología* 2012; **24**: 42–51.
