# Supplementary material for: Stage at diagnosis and stage-specific survival of breast cancer in Latin America and the Caribbean: A systematic review and meta-analysis
Source: PLoS One. 2019 Oct 16;14(10):e0224012. doi: 10.1371/journal.pone.0224012 (PMC6799865; doi:10.1371/journal.pone.0224012)
Supplement: S2 Table — (PDF) [file pone.0224012.s006.pdf]

Quality assessment of studies evaluating stage at diagnosis

1) Minimizing selection bias (max: 12):

1.1) Timing of data collection: Score 0 if unclear; Score 2 if retrospective; Score 4 if prospective.

1.2) Study design (regarding sampling): Score 0 if unclear; Score 1.5 if opportunistic case series/convenience sample; Score 2.5 if consecutive case series/random sample; Score 4 if population-based registry.

1.3) Percentage of overall study population for which information on stage at diagnosis is provided: Score 0 if unclear; Score 2 if < 80% of total cases; Score 4 if ≥ 80% of total cases.

2) Minimizing information bias (max: 12):

2.1) What staging criteria was used? Score 0 if staging criteria was not reported; Score 4 if staging criteria was reported.

2.2) Staging methods: Score 0 if unclear; Score 2 if clinical only; Score 4 if clinical and imaging and/or other complementary exams.

2.3) How were data on stage at presentation reported? Score 0 if unclear; Score 2 if only data for aggregated categories of early (stages I and II combined) and late stage (stages III and IV combined) were given; Score 4 if data provided separately for each one of the four stages (I, II, III and IV).

3) Assessment of other important variables related to stage at diagnosis (max: 5):

3.1) Age at diagnosis (e.g. mean, median or age-categories): Score 0 if not reported; Score 1 if reported.

3.2) Menopausal status at diagnosis: Score 0 if not described; Score 1 if described.

3.3) Year of diagnosis: Score 0 if not reported; Score 1 if reported.

3.4) Tumor grade Score 0 if not reported; Score 1 if reported.

3.5) Hormone receptor and/or HER2 status and/or molecular subtype: Score 0 if not reported; Score 1 if reported.

The overall quality of each study will be expressed as the sum of its item-specific scores. The higher the score attained by a study, the higher the methodological quality, that is, the lower the risk that its findings may have been affected by bias.

S2 Table. Quality assessment of studies included for the outcome of stage at diagnosis

Country Code-Author (year)	1) Minimizing selection bias			2) Minimizing information bias			3) Assessment of other important variables related to stage at presentation					Final score
	**1.1)** Timing of data collection	**1.2)** Study design (regarding sampling)	**1.3)** Percentage of overall study population for which information on stage at diagnosis is provided	**2.1)** What staging criteria was used?	**2.2)** Staging methods	**2.3)** How were data on stage at presentation reported?	**3.1)** Age at diagnosis	**3.2)** Menopausal status	**3.3)** Year of diagnosis	**3.4)** Tumor grade	**3.5)** Hormone receptor and/or HER2 status and/or molecular subtype	
**Caribbean**												
BHS-Mungrue (2016)	2	4	2	4	4	4	1	0	1	1	0	23
BRB-Nemesure (2009)	2	4	0	4	0	4	1	1	1	0	1	18
CUB-Viera-Hernández (2011)	2	2.5	4	4	4	4	1	0	1	0	0	22.5
CUB-Ruiz-Lorente (2010)	2	2.5	4	4	0	4	1	0	1	0	0	18.5
CUB-González-Longoria Boada and Lemes-Báez (2011)	0	4	4	4	0	4	1	0	0	0	0	17
CUB-Garrote (2011)	2	4	4	4	0	4	1	0	0	0	0	19
CUB-Milián-Mosquera (2015)	2	0	4	4	0	4	1	0	0	0	0	15
CUB-Gómez-Delgado (2017)	2	2.5	4	0	0	4	1	0	1	0	0	14.5
HTI-DeGennaro (2018)	2	2.5	4	4	4	4	1	1	1	1	1	25.5
JAM-Alfred (2012)	2	2.5	4	4	0	4	1	1	1	1	1	21.5

Country Code-Author (year)	1) Minimizing selection bias			2) Minimizing information bias			3) Assessment of other important variables related to stage at presentation					Final score
	**1.1)** Timing of data collection	**1.2)** Study design (regarding sampling)	**1.3)** Percentage of overall study population for which information on stage at diagnosis is provided	**2.1)** What staging criteria was used?	**2.2)** Staging methods	**2.3)** How were data on stage at presentation reported?	**3.1)** Age at diagnosis	**3.2)** Menopausal status	**3.3)** Year of diagnosis	**3.4)** Tumor grade	**3.5)** Hormone receptor and/or HER2 status and/or molecular subtype	
PRI-Ortiz (2010)	4	2.5	4	4	0	4	1	0	1	1	1	22.5
TTO-Warner (2015)	2	4	4	4	0	2	1	0	1	1	0	19
TTO-Raju (1989)	2	2.5	4	4	0	4	1	0	0	0	0	17.5
**Central America**												
CRI-Ortiz-Barboza (2011)	0	4	4	0	0	2	1	0	1	0	0	12
HND-Munoz (2011)	2	2.5	4	4	0	4	1	1	1	0	0	19.5
MEX-Pérez-Michel (2009)	2	2.5	4	4	0	4	1	0	0	1	1	19.5
MEX-Arce-Salinas (2012)	0	2.5	4	4	0	4	0	0	1	0	1	16.5
MEX-Lara-Medina (2011)	2	2.5	4	4	0	4	1	1	0	1	1	20.5
MEX-Reynoso-Noverón (2017)	2	2.5	4	4	4	4	1	1	1	1	1	25.5
MEX-Maffuz-Aziz (2016)	2	2.5	4	4	0	4	1	0	0	0	1	18.5
MEX-Ortega-Cervantes (2013)	2	2.5	4	4	0	4	1	0	1	0	0	18.5
MEX-Leon-Rodriguez (2017)	2	2.5	4	4	0	4	1	1	1	0	0	19.5

Country Code-Author (year)	1) Minimizing selection bias			2) Minimizing information bias			3) Assessment of other important variables related to stage at presentation					Final score
	**1.1)** Timing of data collection	**1.2)** Study design (regarding sampling)	**1.3)** Percentage of overall study population for which information on stage at diagnosis is provided	**2.1)** What staging criteria was used?	**2.2)** Staging methods	**2.3)** How were data on stage at presentation reported?	**3.1)** Age at diagnosis	**3.2)** Menopausal status	**3.3)** Year of diagnosis	**3.4)** Tumor grade	**3.5)** Hormone receptor and/or HER2 status and/or molecular subtype	
MEX-Álvarez-Bañuelos (2016)	2	1.5	4	4	0	2	1	1	1	1	1	18.5
MEX-Medina-Franco (2017)	2	2.5	4	4	0	4	0	0	0	0	0	16.5
MEX-Ángeles-Llerenas (2016)	2	1.5	4	4	0	4	1	0	1	0	0	17.5
**South America**												
ARG-Bianco (1985)	2	2.5	4	4	0	4	1	1	0	0	0	18.5
ARG-Juarez (2009)	2	1.5	4	0	0	4	1	0	1	0	0	13.5
ARG-Elizalde (2013)	2	4	4	4	0	4	1	1	0	0	0	20
ARG-Grippo (2015)	2	2.5	4	4	0	4	0	0	0	1	1	18.5
ARG-Meiss (2016)	2	2.5	4	4	0	4	1	1	0	1	1	20.5
ARG-Palazzo (2016)	2	4	2	0	0	2	1	0	1	0	0	12
BRA-Antunes (2015)	2	2.5	4	4	0	4	0	0	1	0	0	17.5
BRA-Medeiros (2015)	2	2.5	4	4	0	4	1	0	1	0	0	18.5

Country Code-Author (year)	1) Minimizing selection bias			2) Minimizing information bias			3) Assessment of other important variables related to stage at presentation					Final score
	**1.1)** Timing of data collection	**1.2)** Study design (regarding sampling)	**1.3)** Percentage of overall study population for which information on stage at diagnosis is provided	**2.1)** What staging criteria was used?	**2.2)** Staging methods	**2.3)** How were data on stage at presentation reported?	**3.1)** Age at diagnosis	**3.2)** Menopausal status	**3.3)** Year of diagnosis	**3.4)** Tumor grade	**3.5)** Hormone receptor and/or HER2 status and/or molecular subtype	
BRA-Thuler and Mendonça (2005)	2	2.5	4	4	0	4	0	0	0	0	0	16.5
CHL-Peralta (1995)	0	2.5	4	4	4	4	1	1	1	0	1	22.5
CHL-Pietro (2011)	2	2.5	4	4	0	2	0	0	1	0	0	15.5
COL-González-Mariño (2005)	2	2.5	4	4	0	4	1	0	1	1	0	19.5
COL-Martínez (2012)	2	2.5	4	4	0	4	1	1	0	1	1	20.5
COL-Pardo (2015)	2	2.5	4	4	0	2	1	0	1	0	0	16.5
COL-Piñeros (2008)	2	2.5	4	4	0	4	1	0	0	0	1	18.5
COL-Robledo-Abad (2005)	2	2.5	4	4	0	4	1	0	1	0	1	19.5
COL-Pardo (2003)	2	2.5	4	4	0	4	1	0	1	0	0	18.5
COL-Angarita (2010)	2	2.5	4	4	0	4	1	1	1	0	1	20.5
COL-González-Mariño (2006)	2	2.5	4	4	0	4	1	0	1	1	0	19.5
COL-Lenis and Esparza (1998)	2	2.5	4	4	0	4	1	1	1	0	0	19.5

Country Code-Author (year)	1) Minimizing selection bias			2) Minimizing information bias			3) Assessment of other important variables related to stage at presentation					Final score
	**1.1)** Timing of data collection	**1.2)** Study design (regarding sampling)	**1.3)** Percentage of overall study population for which information on stage at diagnosis is provided	**2.1)** What staging criteria was used?	**2.2)** Staging methods	**2.3)** How were data on stage at presentation reported?	**3.1)** Age at diagnosis	**3.2)** Menopausal status	**3.3)** Year of diagnosis	**3.4)** Tumor grade	**3.5)** Hormone receptor and/or HER2 status and/or molecular subtype	
COL-Ramiréz-Martinéz (2015)	2	2.5	4	4	0	4	1	0	0	0	1	18.5
COL-Garcia (2012)	2	2.5	4	4	0	4	1	1	0	1	1	20.5
ECU-Cueva and Yepez (2014)	0	4	4	4	0	4	1	0	1	0	0	18
ECU-Cueva and Yepez (2009)	0	4	4	4	0	4	1	0	1	0	0	18
ECU-Martinez (2015)	2	4	4	4	0	4	1	0	1	0	0	20
GUY-Taioli (2010)	2	4	4	0	0	2	1	0	1	0	0	14
GUF-Roué (2016)	4	4	4	4	0	4	1	0	1	1	1	24
PRY-Yoffe de Quiroz (2005)	2	2.5	4	0	0	4	0	0	0	0	0	12.5
PER-Diaz (1999)	2	2.5	4	4	4	4	1	1	0	0	0	22.5
PER-Diaz-Valez (2013)	0	2.5	2	0	0	4	0	0	0	0	0	8.5
PER-Larrea-Fernadez (2016)	2	2.5	4	4	0	4	1	1	1	1	1	21.5
PER-Gutierrez (2008)	2	2.5	2	4	0	4	0	0	0	0	0	14.5
PER-Infanzon (2000)	2	2.5	4	4	0	4	1	0	0	0	0	17.5

Country Code-Author (year)	1) Minimizing selection bias			2) Minimizing information bias			3) Assessment of other important variables related to stage at presentation					Final score
	**1.1)** Timing of data collection	**1.2)** Study design (regarding sampling)	**1.3)** Percentage of overall study population for which information on stage at diagnosis is provided	**2.1)** What staging criteria was used?	**2.2)** Staging methods	**2.3)** How were data on stage at presentation reported?	**3.1)** Age at diagnosis	**3.2)** Menopausal status	**3.3)** Year of diagnosis	**3.4)** Tumor grade	**3.5)** Hormone receptor and/or HER2 status and/or molecular subtype	
SUR-vanLeeuwaarde (2011)	2	4	4	4	0	2	1	0	1	1	1	20
URY-Carnejo (2013)	2	2.5	4	4	4	4	1	0	1	1	1	24.5
URY-Malvasio (2017)	2	2.5	4	4	0	4	1	1	1	1	1	21.5
VEN-Ferri (2012)	2	2.5	4	4	0	4	1	0	0	0	0	17.5
VEN-Rebolledo (2012)	2	2.5	4	4	0	4	1	0	0	1	1	19.5

Study references are given on S3 File.
