# Supplementary material for: Stage at diagnosis and stage-specific survival of breast cancer in Latin America and the Caribbean: A systematic review and meta-analysis
Source: PLoS One. 2019 Oct 16;14(10):e0224012. doi: 10.1371/journal.pone.0224012 (PMC6799865; doi:10.1371/journal.pone.0224012)
Supplement: S3 Table — (PDF) [file pone.0224012.s007.pdf]

# Quality assessment of studies evaluating survival probability

**1) Minimizing selection bias (max: 12):**

1.1) Timing of data collection: Score 0 if unclear; Score 2 if retrospective; Score 4 if prospective.

1.2) Study design (regarding sampling): Score 0 if unclear; Score 1.5 if opportunistic case series/convenience sample; Score 2.5 if consecutive case series/random sample; Score 4 if population-based registry.

1.3) Left-truncation: Is it possible that patients may have died before inclusion in the study? Score 0 if unclear (studies do not describe what date was used as reference for survival analysis); Score 1 if left truncation was not avoided (by design or analysis). E.g.: The study included only patients who underwent surgery and evaluated survival from diagnosis or from an unspecified date; Score 4 if left truncation was avoided (by design or analysis). E.g.: All diagnosed patients were included in the survival analysis considered the date of diagnosis, or the study included only patients who underwent surgery were survival analysis considered the date of surgery.

**2) Minimizing information bias (max: 12):**

2.1) Vital status assignment Score 0 if unclear; Score 2 if passive; Score 4 if passive with active component or active.

2.2) Failure definition: Score 0 if unclear; Score 4 if reported (all-cause mortality, disease related mortality, analysis of relative survival).

2.3) Survival analysis method: Score 0 if unclear; Score 4 if reported (actuarial, Kaplan-Meier, relative survival).

**3) Assessment of other important variables related to survival analysis (max: 5):**

3.1) Treatment received: Score 0 if not described; Score 1 if described.

3.2) Age at diagnosis (e.g. mean, median or age-categories): Score 0 if not reported; Score 1 if reported.

3.3) Year of diagnosis: Score 0 if not reported; Score 1 if reported.

3.4) Tumor grade Score 0 if not reported; Score 1 if reported.

3.5) Hormone receptor and/or HER2 status and/or molecular subtype: Score 0 if not reported; Score 1 if reported.

The overall quality of each study will be expressed as the sum of its item-specific scores. The higher the score attained by a study, the higher the methodological quality, that is, the lower the risk that its findings may have been affected by bias.

## S3 Table. Quality assessment of studies included for the outcome of survival probability

| Country Code-Author (year) | 1) Minimizing selection bias | | | 2) Minimizing information bias | | | 3) Assessment of other important variables related to survival analysis | | | | | Final score |
|---|---|---|---|---|---|---|---|---|---|---|---|---|
| | **1.1)** Timing of data collection | **1.2)** Study design (regarding sampling) | **1.3)** Left-truncation | **2.1)** Vital status assignment | **2.2)** Failure definition | **2.3)** Survival analysis method | **3.1)** Age at presentation | **3.2)** Treatment received | **3.3)** Year of diagnosis: | **3.4)** Tumor grade | **3.5)** Hormone receptor and/or HER2 status and/or molecular subtype: | |
| **Caribbean** | | | | | | | | | | | | |
| CUB-Diaz (2004) | 2 | 0 | 0 | 0 | 0 | 4 | 1 | 1 | 1 | 0 | 0 | 9 |
| CUB-Moreno (1998) | 2 | 0 | 0 | 0 | 0 | 0 | 1 | 1 | 0 | 0 | 0 | 4 |
| CUB-Ricardo-Ramirez (2013) | 2 | 0 | 0 | 0 | 0 | 4 | 0 | 0 | 0 | 0 | 0 | 6 |
| CUB- González-Longoria Boada and Lemes-Báez (2011) | 0 | 4 | 4 | 2 | 4 | 4 | 1 | 1 | 0 | 0 | 0 | 20 |
| CUB-Garrote (2011) | 2 | 4 | 1 | 2 | 0 | 4 | 1 | 0 | 0 | 0 | 0 | 14 |
| CUB-Fernández-Garrote (1998) | 2 | 4 | 4 | 2 | 0 | 4 | 1 | 0 | 0 | 0 | 0 | 17 |
| HTI-DeGennaro (2018) | 2 | 2.5 | 4 | 2 | 0 | 0 | 1 | 1 | 1 | 1 | 1 | 15.5 |
| **Central America** | | | | | | | | | | | | |
| CRI-Quiros-Alpizar (2017) | 2 | 2.5 | 0 | 2 | 0 | 4 | 1 | 1 | 1 | 1 | 1 | 15.5 |
| CRI-Ortiz-Barboza (2011) | 0 | 4 | 1 | 2 | 0 | 4 | 1 | 0 | 1 | 0 | 0 | 13 |

| Country Code-Author (year) | 1) Minimizing selection bias | | | 2) Minimizing information bias | | | 3) Assessment of other important variables related to survival analysis | | | | | Final score |
|---|---|---|---|---|---|---|---|---|---|---|---|---|
| | **1.1)** Timing of data collection | **1.2)** Study design (regarding sampling) | **1.3)** Left-truncation | **2.1)** Vital status assignment | **2.2)** Failure definition | **2.3)** Survival analysis method | **3.1)** Age at presentatio n | **3.2)** Treatment received | **3.3)** Year of diagnosis: | **3.4)** Tumor grade | **3.5)** Hormone receptor and/or HER2 status and/or molecular subtype: | |
| EX-Flores-Luna (2008) | 2 | 2.5 | 1 | 0 | 4 | 4 | 1 | 1 | 0 | 1 | 0 | 16.5 |
| MEX-Álvarez-Bañuelos (2016) | 2 | 1.5 | 1 | 2 | 4 | 4 | 1 | 1 | 1 | 1 | 1 | 19.5 |
| MEX-Ángeles-Llerenas (2016) | 4 | 1.5 | 4 | 2 | 4 | 4 | 1 | 0 | 1 | 0 | 0 | 21.5 |
| MEX-Lara-Medina (2011) | 2 | 2.5 | 4 | 0 | 4 | 4 | 1 | 1 | 0 | 1 | 1 | 20.5 |
| MEX-Reynoso-Noverón (2017) | 2 | 2.5 | 4 | 0 | 4 | 4 | 1 | 1 | 1 | 1 | 1 | 21.5 |
| MEX-di Filippo-Echeverri (2004) | 2 | 0 | 1 | 0 | 0 | 4 | 1 | 1 | 1 | 1 | 0 | 11 |
| MEX-Maffuz-Aziz (2016) | 2 | 2.5 | 0 | 0 | 0 | 4 | 1 | 0 | 0 | 0 | 1 | 10.5 |
| MEX-Ramirez-Torres (2016) | 2 | 2.5 | 1 | 0 | 4 | 4 | 1 | 1 | 0 | 1 | 1 | 17.5 |
| MEX-Leon-Rodriguez (2017) | 2 | 2.5 | 0 | 0 | 0 | 4 | 1 | 1 | 1 | 0 | 0 | 11.5 |
| **South America** | | | | | | | | | | | | |
| ARG-Iturbe (2011) | 2 | 1.5 | 4 | 0 | 4 | 0 | 1 | 1 | 1 | 0 | 1 | 15.5 |

| Country Code-Author (year) | 1) Minimizing selection bias | | | 2) Minimizing information bias | | | 3) Assessment of other important variables related to survival analysis | | | | | Final score |
|---|---|---|---|---|---|---|---|---|---|---|---|---|
| | **1.1)** Timing of data collection | **1.2)** Study design (regarding sampling) | **1.3)** Left-truncation | **2.1)** Vital status assignment | **2.2)** Failure definition | **2.3)** Survival analysis method | **3.1)** Age at presentation | **3.2)** Treatment received | **3.3)** Year of diagnosis: | **3.4)** Tumor grade | **3.5)** Hormone receptor and/or HER2 status and/or molecular subtype: | |
| ARG-Arce (2013) | 2 | 2.5 | 0 | 4 | 4 | 4 | 0 | 1 | 1 | 0 | 0 | 18.5 |
| ARG-Berra (2016) | 2 | 1.5 | 4 | 0 | 0 | 4 | 1 | 1 | 0 | 1 | 1 | 15.5 |
| BRA-Stival (2012) | 2 | 0 | 0 | 0 | 4 | 4 | 1 | 0 | 1 | 1 | 1 | 14 |
| BRA-Ayala (2012) | 2 | 1.5 | 4 | 2 | 4 | 4 | 1 | 0 | 1 | 0 | 0 | 19.5 |
| BRA-Guerra (20090 | 2 | 2.5 | 1 | 4 | 4 | 4 | 1 | 1 | 1 | 0 | 0 | 20.5 |
| BRA-Schneider (2009) | 2 | 2.5 | 4 | 2 | 4 | 4 | 1 | 1 | 1 | 0 | 0 | 21.5 |
| BRA-Moraes (2006) | 2 | 2.5 | 4 | 2 | 4 | 4 | 1 | 1 | 1 | 1 | 1 | 23.5 |
| BRA-Vazquez (2016) | 2 | 2.5 | 4 | 0 | 4 | 4 | 1 | 1 | 0 | 1 | 1 | 20.5 |
| BRA-Fayer (2016) | 2 | 2.5 | 0 | 4 | 4 | 4 | 1 | 1 | 1 | 0 | 1 | 20.5 |
| BRA-Carrara (2017) | 2 | 2.5 | 4 | 0 | 0 | 4 | 1 | 1 | 1 | 1 | 1 | 17.5 |
| CHL-Peralta (1995) | 0 | 2.5 | 0 | 0 | 0 | 4 | 1 | 1 | 1 | 0 | 1 | 10.5 |
| CHL-Jurgensen (2009) | 2 | 2.5 | 0 | 0 | 0 | 4 | 1 | 0 | 1 | 0 | 0 | 10.5 |
| CHL-Acevedo (2006) | 2 | 2.5 | 0 | 0 | 0 | 4 | 1 | 1 | 1 | 0 | 0 | 11.5 |

| Country Code-Author (year) | 1) Minimizing selection bias | | | 2) Minimizing information bias | | | 3) Assessment of other important variables related to survival analysis | | | | | Final score |
|---|---|---|---|---|---|---|---|---|---|---|---|---|
| | **1.1)** Timing of data collection | **1.2)** Study design (regarding sampling) | **1.3)** Left-truncation | **2.1)** Vital status assignment | **2.2)** Failure definition | **2.3)** Survival analysis method | **3.1)** Age at presentation | **3.2)** Treatment received | **3.3)** Year of diagnosis: | **3.4)** Tumor grade | **3.5)** Hormone receptor and/or HER2 status and/or molecular subtype: | |
| COL-Robledo-Abad (2005) | 2 | 2.5 | 1 | 0 | 4 | 4 | 1 | 1 | 1 | 0 | 1 | 17.5 |
| COL-Ospino (2010) | 2 | 2.5 | 4 | 2 | 4 | 4 | 1 | 1 | 1 | 1 | 1 | 23.5 |
| COL-Ospino (2011) | 2 | 2.5 | 4 | 2 | 4 | 4 | 1 | 1 | 1 | 1 | 1 | 23.5 |
| COL-Zuluaga-Liberato (2016) | 2 | 2.5 | 0 | 4 | 4 | 4 | 1 | 1 | 0 | 1 | 1 | 20.5 |
| ECU-Jorge (1994) | 2 | 2.5 | 0 | 0 | 0 | 4 | 1 | 1 | 1 | 0 | 0 | 11.5 |
| PER-Diaz (1999) | 2 | 2.5 | 4 | 4 | 4 | 4 | 1 | 1 | 0 | 0 | 0 | 22.5 |
| PER-Larrea-Fernadez (2016) | 2 | 2.5 | 4 | 0 | 0 | 4 | 1 | 1 | 1 | 1 | 1 | 17.5 |
| URY-Vazquez (2005) | 2 | 0 | 0 | 0 | 4 | 4 | 1 | 1 | 0 | 0 | 1 | 13 |
| VEN-Hung (2012) | 2 | 2.5 | 4 | 0 | 4 | 4 | 1 | 0 | 1 | 1 | 1 | 20.5 |
| VEN-Godoy (2000) | 2 | 2.5 | 0 | 0 | 0 | 4 | 1 | 1 | 0 | 1 | 1 | 12.5 |
| VEN-Pacheco-Soler (2000) | 2 | 0 | 0 | 0 | 0 | 4 | 1 | 1 | 0 | 1 | 0 | 9 |
| VEN-Ravelo-Celis (2007) | 0 | 2.5 | 0 | 0 | 0 | 4 | 1 | 0 | 0 | 0 | 1 | 8.5 |
| VEN-Acosta-Marin (2011) | 2 | 0 | 0 | 0 | 0 | 4 | 1 | 1 | 0 | 0 | 0 | 8 |

| Country Code-Author (year) | 1) Minimizing selection bias | | | 2) Minimizing information bias | | | 3) Assessment of other important variables related to survival analysis | | | | | Final score |
|---|---|---|---|---|---|---|---|---|---|---|---|---|
| | **1.1)** Timing of data collection | **1.2)** Study design (regarding sampling) | **1.3)** Left-truncation | **2.1)** Vital status assignment | **2.2)** Failure definition | **2.3)** Survival analysis method | **3.1)** Age at presentation | **3.2)** Treatment received | **3.3)** Year of diagnosis: | **3.4)** Tumor grade | **3.5)** Hormone receptor and/or HER2 status and/or molecular subtype: | |
| VEN-Vera (2002) | 2 | 2.5 | 0 | 0 | 0 | 4 | 1 | 1 | 0 | 0 | 0 | 10.5 |

Study references are given on S3 File.
