# Supplementary material for: Stage at diagnosis and stage-specific survival of breast cancer in Latin America and the Caribbean: A systematic review and meta-analysis
Source: PLoS One. 2019 Oct 16;14(10):e0224012. doi: 10.1371/journal.pone.0224012 (PMC6799865; doi:10.1371/journal.pone.0224012)
Supplement: S4 Table — (PDF) [file pone.0224012.s008.pdf]

S4 Table. Characteristics of the studies included for the outcome of stage at diagnosis

Country Code-Author (year)	Patients with breast cancer	Patients with known breast cancer stage (%)	Age*	Year of diagnosis	Sampling/ Study type	Country/ Province/ City	Location	Type of facility	Staging system	Staging method	Quality score
**Caribbean**											
BHS-Mungrue (2016)	270	134 (49.6)	56.6 ± 13.8‡	2009-2011	Population-based/ Case series	Bahamas	National Oncology Board	Public	TNM	Clinical and imaging	23
BRB-Nemesure (2009)	222	222 (100)	56.8 ± 14.3‡	2002-2006	Population-based/ Case series	Barbados	Barbados National Cancer Study	Public	TNM	NR	18
CUB-Viera-Hernández (2011)	156	141 (90.4)	≥60†	2000-2010	Consecutive/ Case series	Isla de la Juventud	1 center (Hospital General Docente Héroes del Baire)	Public	TNM (6th ed.)	Clinical and imaging	22,5
CUB-Ruiz-Lorente (2010)	128	128 (100)	≥40 <60†	2006-2009	Consecutive/ Case series	Havana	1 center (Servicio de Oncología del Hospital Ramón González Coro)	Public	TNM	NR	18,5
CUB-González-Longoria Boada (2011)	171	170 (99.4)	58.2‡	1997-1998	Population-based/ Cohort	Granma province	Registro Nacional de Cáncer	Public	TNM (5th ed.)	NR	17
CUB-Garrote (2011)	2169	1772 (81.7)	≥40 <60†	1994-1995	Population-based/ Cohort	Cuba	Registro Nacional de Cáncer	Public	TNM	NR	19
CUB-Milián-Mosquera (2015)	59	54 (91.5)	≥40 <60†	2013	Unclear/ Case series	Las Tunas	NR	Public	TNM	NR	15
CUB-Gómez-Delgado (2017)	1315	1315 (100)	(24-97)	2010-2014	Consecutive/ Case series	Santa Clara	1 center (Hospital Universitario Dr. Celestino Hernández Robau)	Public	NR	NR	14,5

Country Code-Author (year)	Patients with breast cancer	Patients with known breast cancer stage (%)	Age*	Year of diagnosis	Sampling/ Study type	Country/ Province/ City	Location	Type of facility	Staging system	Staging method	Quality score
HTI-DeGennaro (2018)	525	445 (84.8)	59 ± 13‡	2013-2017	Consecutive/ Cohort	Port-au-Prince	1 center (started at Hospital Bernard Mevs, then moved to St Luke's Hospital)	Private	TNM (6th ed.)	Clinical and imaging	25,5
JAM-Alfred (2012)	199	184 (92.5)	≥40 <60†	2002-2009	Consecutive/ Case series	Kingston	1 center (University Hospital of the West Indies)	Public	TNM	NR	21,5
PRI-Ortiz (2010)	985	867	58.3 ± 14‡	2000-2005	Consecutive/ Case series	San Juan	2 centers (González Martínez Oncologic Hospital and Auxilio Mutuo Hospital)	Private	SEER Summary Staging 2002	NR	22,5
TTO-Warner (2015)	3767	3095 (82.2)	56.6 ± 14.6‡	1995-2007	Population-based/ Case series	Trinidad and Tobago	Cancer Registry of Trinidad and Tobago	Public and private	NR	NR	19
TTO-Raju and Naraynsingh (1989)	363	363 (100)	(23-92)	1976-1980	Consecutive/ Case series	Port of Spain	1 center (Port of Spain General Hospital)	Public	Manchester	NR	17,5
**Central America**											
CRI-Ortiz-Barboza (2011)	2462	2105 (85.5)	≥40 <60†	1995-2000	Population-based/ Cohort	Costa Rica	National tumour registry	Public and private	TNM	NR	12
HND-Muñoz (2011)	685	653 (95.3)	(22-102)	1999-2009	Consecutive/ Case series	Tegucigalpa	1 center (Centro de Cáncer Emma Romero Callejas)	NR	TNM	NR	19,5
MEX-Pérez-Michel (2009)	397	397 (100)	≥40 <60†	2002-2007	Consecutive/ Case series	Obregón	1 center (Centro Médico Nacional del Noroeste, Instituto Mexicano del Seguro Social)	Public	NR	NR	19,5

Country Code-Author (year)	Patients with breast cancer	Patients with known breast cancer stage (%)	Age*	Year of diagnosis	Sampling/ Study type	Country/ Province/ City	Location	Type of facility	Staging system	Staging method	Quality score
MEX-Álvarez-Bañuelos (2016)	114	104 (91.2)	≥40 <60†	2009	Consecutive/ Cohort	Xalapa	1 center (Centro Estatal de Cancerología)	Public	TNM (7th ed.)	NR	18,5
MEX-Arce-Salinas (2012)	1247	1132 (90.8)	≥40 <60†	2008-2009	Consecutive/ Case series	Mexico city	1 center (Instituto Nacional de Cancerología)	Public	TNM	NR	16,5
MEX-Ángeles-Llerenas (2016)	854	816 (95.6)	51 (IQR= 44.2-60.3)	2007-2009	Convenience/ Cohort	Mexico	11 centers	Public	TNM	NR	17,5
MEX-Lara-Medina (2011)	2074	2074 (100)	50 (19-96)	1998-2008	Consecutive/ Cohort'	México City	1 center (Instituto Nacional de Cancerología)	Public	TNM (6th ed.)	NR	20,5
MEX-Reynoso-Noverón (2017)	4300	4300 (100)	52 ± 12.1‡	2007-2013	Consecutive/ Cohort	México City	1 center (Instituto Nacional de Cancerología)	Public	TNM	Clinical and imaging	25,5
MEX-Maffuz-Aziz (2016)	4902	4361 (89.0)	53.7 ± 12.2‡	2005-2014	Consecutive/ Cohort	México City	1 center (Instituto de Enfermedades de la Mama)	Public	TNM (7th ed.)	NR	18,5
MEX-Ortega-Cervantes (2013)	406	358 (88.2)	(25-85)	2006-2010	Consecutive/ Case series	Tepic	1 center (Centro Estatal de Cancerología)	Public	TNM (7th ed.)	NR	18,5
MEX-Leon-Rodriguez (2017)	291	250 (85.9)	57 (27-89)	2000-2016	Consecutive/ Cohort	Mexico City	1 center (National Institute of Health Sciences and Nutrition Salvador Zubiran)	Public	TNM	NR	19,5
MEX-Medina-Franco (2017)	230	186 (80.9)	≥40 <60†	2001-2016	Consecutive/ Case series	México City	1 center (Hospital Médica Sur)	Private	TNM	NR	16,5
**South America**											

Country Code-Author (year)	Patients with breast cancer	Patients with known breast cancer stage (%)	Age*	Year of diagnosis	Sampling/ Study type	Country/ Province/ City	Location	Type of facility	Staging system	Staging method	Quality score
ARG-Bianco (1985)	1658	1650 (99.5)	56.1	1983-1984	Consecutive/ Case series	Argentina	86 centers	Public and private	TNM	NR	18,5
ARG-Juarez (2009)	307	281 (91.5)	≥40 <60†	1998-2003	Convenience/ Case series	Cordoba	4 centers (Hospital Nacional de Clínicas; Maternidad Nacional; Maternidad Provincial; and Hospital San Roque)	Public	NR	NR	13,5
ARG-Elizalde (2013)	4041	3383 (83.7)	57.7±13.2‡	2010-2012	Consecutive/ Case series	Buenos Aires and La Plata	16 centers	NR	TNM (7th ed.)	NR	20
ARG-Grippo (2015)	303	303 (100)	NR	2008-2013	Consecutive/ Case series	General Roca	1 center (Unidad Integral de Oncología)	NR	TNM	NR	18,5
ARG-Meiss (2016)	1732	1470 (84.9)	59 (23-92)	2012-2014	Consecutive/ Case series	Argentina	81 centers	NR	TNM	NR	20,5
ARG-Palazzo (2016)	413	257 (62.2)	Public: 55.3 ± 13.1‡ Private:: 61.8 ± 12.7‡	2013	Population-based/ Case series	Partido de General Pueyrredón	Multicentric (Registro de Cáncer de Base Poblacional)	Public and private	TNM (6th ed.)	NR	12
BRA-Antunes (2015)	152	133 (87.5)	≥60†	2007-2011	Consecutive/ Case series	São Paulo city	1 center (Hospital Israelita Albert Einstein)	Public and private	TNM	NR	17,5
BRA-Medeiros (2015)	137593	113877 (82.8)	54 (18-80)	2000-2011	Consecutive/ Case series	Brazil	239 centers (Registros Hospitalares de Câncer)	Public	TNM (6th ed.	NR	18,5

Country Code-Author (year)	Patients with breast cancer	Patients with known breast cancer stage (%)	Age*	Year of diagnosis	Sampling/ Study type	Country/ Province/ City	Location	Type of facility	Staging system	Staging method	Quality score
BRA-Thuler and Mendonça (2005)	50900	40879 (80.3)	NR	1990-2002	Consecutive/ Case series	Brazil	Registros Hospitalares de Câncer<br>1990-1994: 18 centers<br>1995-2002: 96 centers	Public	TNM	NR	16,5
CHL-Peralta (1995)	357	357 (100)	(22-92)	1985-1995	Consecutive/ Cohort	Región Metropolitana de Chile	Multicentric (Servicio de Salud Metropolitano Central)	Public	TNM	Clinical and imaging	22,5
CHL-Prieto (2011)	23000	21120 (91.8)	NR	2000-2009	Consecutive/ Case series	Chile	29 centers (Sistema Nacional de Servicios de Salud)	Public	TNM	NR	15,5
COL-González-Mariño (2005)	207	187 (90.3)	54 (31-85)	2003	Consecutive/ Case series	Bogota	1 center (Clínica San Pedro Claver)	Public	TNM	NR	19,5
COL-Martínez (2012)	308	299 (97.1)	55.2 ± 12.6‡	2005-2009	Consecutive/ Case series	Ibaqué	1 center (Hospital Federico Lleras Acosta)	Public	TNM	NR	20,5
COL-Pardo (2015)	169	135 (86.8)	55‡	2006-2008	Consecutive/ Case series	Villavicencio	1 center (Unidad de Cáncer del Hospital Departamental de Villavicencio)	Public	TNM	NR	16,5
COL-Piñeros (2008)	1106	1004 (90.8)	53.4	2006	Consecutive/ Case series	Bogota	15 centers	NR	TNM	NR	18,5
COL-Robledo-Abad (2005)	1328	1216 (91.6)	53 (25-92)	1989-2003	Consecutive/ Cohort	Bogota	1 center (Unidad Oncológica del Country)	Private	TNM (5th ed.)	NR	19,5
COL-Pardo (2003)	608	528 (86.8)	NR	2002	Consecutive/ Case series	Bogota	1 center (Instituto Nacional de Cancerología)	Public	TNM	NR	18,5

Country Code-Author (year)	Patients with breast cancer	Patients with known breast cancer stage (%)	Age*	Year of diagnosis	Sampling/ Study type	Country/ Province/ City	Location	Type of facility	Staging system	Staging method	Quality score
COL-Angarita (2010)	232	216 (93.1)	55 ± 13.5‡	2004-2007	Consecutive/ Case series	Bogota	1 center (Clínica de Seno y Tejidos Blandos del Centro Javeriano de Oncología, Hospital Universitário San Ignacio)	Private	TNM	NR	20,5
COL-González-Mariño (2006)	220	193 (87.7)	59‡	2004	Consecutive/ Case series	Bogota	1 center (Clínica San Pedro Claver)	NR	TNM	NR	19,5
COL-Lenis and Esparza (1998)	296	281 (94.9)	52.6 ± 13.4‡	1984-1996	Consecutive/ Case series	Manizales	1 center (Hospital de Caldas)	Public	TNM	NR	19,5
COL-Ramiréz-Martinéz (2015)	1480	1262 (85.3)	54‡	2006-2013	Consecutive/ Case series	Medellín	1 center	NR	TNM	NR	18,5
COL-García (2012)	84	84 (100)	54.2 ± 11.8‡	2006-2008	Consecutive/ Case series	Bogota	2 centers (Instituto Nacional de Cancerología and Hospital de San José)	Public	TNM	NR	20,5
ECU-Cueva and Yepez (2014)	1398	1157 (82.8)	≥40 <60†	2006-2010	Population-based	Quito	Registro Nacional de Tumores	Public and private	TNM	NR	18
ECU-Cueva and Yepez (2009)	732	620 (84.7)	≥40 <60†	2003-2005	Population-based	Quito	Registro Nacional de Tumores	Public and private	TNM	NR	18
ECU-Martínez (2015)	308	302 (98.1)	≥40 <60†	2005-2009	Population-based	Cuenca	Registro Nacional de Tumores	Public and private	TNM	NR	20
GUY-Taioli (2010)	499	445 (89.2)	53.1 ± 12.7‡	1995-2007	Population-based	Guyana	Guyana Registry	NR	NR	NR	14

Country Code-Author (year)	Patients with breast cancer	Patients with known breast cancer stage (%)	Age*	Year of diagnosis	Sampling/ Study type	Country/ Province/ City	Location	Type of facility	Staging system	Staging method	Quality score
GUF-Roué (2016)	269	239 (88.8)	52 (27-94)	2003-2009	Population-based	French Guiana	Cancer Registry of French Guiana	Public and private	TNM	NR	24
PRY-Yoffe de Quiroz (2005)	80	80 (100)	≥40 <60†	2004- 2005	Consecutive/ Case series	Asunsión	1 center (Departamento de Oncología del Hospital de Clínicas, Facultad de Ciencias Médicas UNA)	Public	NR	NR	12,5
PER-Díaz (1999)	279	263 (94.3)	≥40 <60†	1966-1995	Consecutive/ Cohort	Trujillo	1 center (Hospital Belén)	Public	TNM (5th ed.)	Clinical and imaging	22,5
PER-Díaz-Vélez (2013)	742	545 (73.5)	NR	2007-2012	Consecutive/ Case series	Lambayeque	multicentric (Registro Hospitalario de cáncer de EsSalud)	Public	NR	NR	8,5
PER-Larrea-Fernández (2016)	75	75 (100)	42 (27-49)	2009-2010	Consecutive/ Cohort	Lima	1 center (Hospital Nacional Guillermo Almenara Irigoyen)	Public	TNM (7th ed.)	NR	21,5
PER-Gutiérrez and Alarcón (2008)	2956	1505 (50.9)	NR	2000-2004	Consecutive/ Case series	Lima	1 center (Instituto Nacional de Enfermedades Neoplásicas)	Public	TNM (6th ed.)	NR	14,5
PER-Infanzón (2000)	126	126 (100)	76,1‡	1990-1995	Consecutive/ Case series	Lima	1 center (Servicio de Oncología Ginecológica de Hospital Nacional Edgardo Rebagliati Martins de EsSalud)	Public	TNM	NR	17,5
SUR-van Leeuwaarde (2011)	419	351 (83.8)	55 (26-90)	1994-2003	Population-based/ Case series	Surinam	Registry	Public and private	TNM	NR	20

Country Code-Author (year)	Patients with breast cancer	Patients with known breast cancer stage (%)	Age*	Year of diagnosis	Sampling/ Study type	Country/ Province/ City	Location	Type of facility	Staging system	Staging method	Quality score
URY-Camejo (2013)	115	109 (94.8)	63 (35-89)	2009-2014	Consecutive/ Case series	Montevideo	1 center (Unidad Docente Asistencial de Mastología)	Public	TNMp	Clinical and imaging	24,5
URY-Malvasio (2017)¤	107	107 (100)	(24-39)	2006-2012	Consecutive/ Case series	Montevideo	3 centers (Servicio de Oncología del Hospital de Clínicas; Centro Hospitalario Pereira Rossell; and Centro de Asistencia del Sindicato Médico del Uruguay- Institución de Asistencia Médica Privada de Profesionales sin fines de lucro)	Public and private	TNM	NR	21,5
VEN-Ferri (2012)	446	411 (92.2)	(22- 91)	1990-2009	Consecutive/ Case series	Valencia	1 center (Unidad de Mastología, Centro Médico Dr. Rafael Guerra Méndez)	Private	TNM (6th ed.)	NR	17,5
VEN-Rebolledo (2012)	179	179 (100)	(28-87)	1999-2007	Consecutive/ Case series	Valencia	1 center (Hospital Metropolitano del Norte)	Private	TNM	NR	19,5

ed., Edition; NR, Not reported; TNM, tumor, Lymph Node, Metastasis staging system; SEER, Surveillance, Epidemiology, and End Results Program
* Age is given as median (range) unless otherwise indicated.
‡ mean age ± standard deviation.
† studies provided age as the distribution among age groups; the indicated age group includes most participants.
¤ URY-Malvasio (2017) analyzed women younger than 40 years old. Study references are given on S3 File.
