# Supplementary material for: Stage at diagnosis and stage-specific survival of breast cancer in Latin America and the Caribbean: A systematic review and meta-analysis
Source: PLoS One. 2019 Oct 16;14(10):e0224012. doi: 10.1371/journal.pone.0224012 (PMC6799865; doi:10.1371/journal.pone.0224012)
Supplement: S5 Table — (PDF) [file pone.0224012.s009.pdf]

# S5 Table. Percentage of patients diagnosed at each stage

| Country Code-Author (year) | Stage at diagnosis (%) | | | | | | | | |
|---|---|---|---|---|---|---|---|---|---|
| | I | I-IIa | I-II | II | I-III | IIb-III | III | III-IV | IV |
| **Caribbean** | | | | | | | | | |
| TTO-Raju and Naraynsingh (1989) | 51.0 | - | - | 34.0 | - | - | 10.0 | - | 5.0 |
| CUB-Milián-Mosquera (2015) | 24.1 | - | - | 46.3 | - | - | 24.1 | - | 5.6 |
| JAM-Alfred (2012) | 9.8 | - | - | 47.3 | - | - | 34.8 | - | 8.2 |
| CUB-Viera-Hernández (2011) | 12.8 | - | - | 35.5 | - | - | 39.0 | - | 12.8 |
| CUB-Gómez-Delgado (2017) | 24.6 | - | - | 2.0 | - | - | 54.8 | - | 18.6 |
| BHS-Mungrue (2016) (C) | - | 44.1 | - | - | - | 54.2 | - | - | 1.7 |
| CUB-González-Longoria Boada (2011) | - | 34.1 | - | - | - | 62.9 | - | - | 2.9 |
| CUB-Ruiz-Lorente (2010) | - | 32.9 | - | - | - | 62.5 | - | - | 4.6 |
| BHS-Nemesure (2009) | - | 54.5 | - | - | - | 39.2 | - | - | 6.3 |
| BHS-Mungrue (2016) (B) | - | 51.7 | - | - | - | 41.4 | - | - | 6.9 |
| BHS-Mungrue (2016) (A) | - | 42.3 | - | - | - | 47.9 | - | - | 9.9 |
| HTI-DeGennaro (2018) | - | 6.1 | - | - | - | 65.4 | - | - | 28.5 |
| PRI-Ortiz (2010) | - | - | 64.0 | - | - | - | 33.8 | - | 2.2 |
| CUB-Garrote (2011) | - | - | 53.2 | - | - | - | 40.8 | - | 6.0 |
| TTO-Warner (2015) | - | - | 48.6 | - | - | - | 41.0 | - | 10.4 |
| **Central America** | | | | | | | | | |
| MEX-Pérez-Michel (2009) | 32.2 | - | - | 38.3 | - | - | 26.7 | - | 2.8 |
| MEX-Ángeles-Llerenas (2016) | 10.8 | - | - | 39.7 | - | - | 41.9 | - | 7.6 |
| MEX-Lara-Medina (2011) | 9.7 | - | - | 34.6 | - | - | 44.0 | - | 11.7 |
| MEX-Reynoso-Noverón (2017) | 14.2 | - | - | 36.6 | - | - | 36.2 | - | 13.0 |
| MEX-Ortega-Cervantes (2013) (A) | - | 21.9 | - | - | - | 75.0 | - | - | 3.1 |
| MEX-Medina-Franco (2017) | - | 79.0 | - | - | - | 17.7 | - | - | 3.2 |
| MEX-Ortega-Cervantes (2013) (C) | - | 29.8 | - | - | - | 64.8 | - | - | 5.4 |
| MEX-Leon-Rodriguez (2017) | - | 66.0 | - | - | - | 28.4 | - | - | 5.6 |
| MEX-Ortega-Cervantes (2013) (B) | - | 30.0 | - | - | - | 63.5 | - | - | 6.5 |
| MEX-Maffuz-Aziz (2016) | - | 41.5 | - | - | - | 50.6 | - | - | 7.9 |
| MEX-Arce-Salinas (2012) (A) | - | 23.0 | - | - | - | 65.4 | - | - | 11.7 |
| MEX-Ortega-Cervantes (2013) (E) | - | 17.1 | - | - | - | 71.0 | - | - | 11.9 |

| Country Code-Author (year) | Stage at diagnosis (%) | | | | | | | | |
|---|---|---|---|---|---|---|---|---|---|
| | I | I-IIa | I-II | II | I-III | IIb-III | III | III-IV | IV |
| MEX-Ortega-Cervantes (2013) (D) | - | 34.7 | - | - | - | 53.0 | - | - | 12.3 |
| HND-Muñoz (2011) (A) | - | 34.9 | - | - | - | 52.4 | - | - | 12.7 |
| MEX-Arce-Salinas (2012) (B) | - | 23.5 | - | - | - | 63.0 | - | - | 13.6 |
| HND-Muñoz (2011) (C) | - | 34.4 | - | - | - | 51.5 | - | - | 14.1 |
| HND-Muñoz (2011) (B) | - | 30.9 | - | - | - | 51.6 | - | - | 17.6 |
| MEX-Álvarez-Bañuelos (2016) | - | - | 50.0 | - | - | - | - | 50.0 | - |
| **South America** | | | | | | | | | |
| ARG-Elizalde (2013) | 45.9 | - | - | 41.4 | - | - | 11.6 | - | 1.1 |
| VEN-Rebolledo (2012) | 5.6 | - | - | 59.2 | - | - | 34.1 | - | 1.1 |
| VEN-Ferri (2012) | 27.3 | - | - | 54.0 | - | - | 17.5 | - | 1.2 |
| PER-Larrea-Fernández (2016) | 14.7 | - | - | 48.0 | - | - | 34.7 | - | 2.7 |
| ARG-Bianco (1985) | 30.7 | - | - | 43.6 | - | - | 22.1 | - | 3.5 |
| ARG-Grippo (2015) | 36.0 | - | - | 43.9 | - | - | 16.5 | - | 3.6 |
| URY-Malvasio (2017) | 21.5 | - | - | 43.9 | - | - | 30.8 | - | 3.7 |
| BRA-Antunes (2015) | 48.9 | - | - | 29.3 | - | - | 16.5 | - | 5.3 |
| CHL-Peralta (1995) | 9.0 | - | - | 49.6 | - | - | 35.0 | - | 6.4 |
| ECU-Martínez (2015) | 18.2 | - | - | 51.0 | - | - | 23.5 | - | 7.3 |
| URY-Camejo (2013) | 24.8 | - | - | 45.9 | - | - | 22.0 | - | 7.3 |
| PER-Díaz-Vélez (2013) | 7.2 | - | - | 51.9 | - | - | 33.0 | - | 7.9 |
| BRA-Medeiros (2015) | 18.1 | - | - | 40.8 | - | - | 31.9 | - | 9.1 |
| ECU-Cueva and Yepez (2014) | 21.1 | - | - | 45.3 | - | - | 22.1 | - | 11.6 |
| PER-Gutiérrez and Alarcón (2008) | 6.4 | - | - | 42.9 | - | - | 39.1 | - | 11.6 |
| ARG-Juarez (2009) | 20.6 | - | - | 35.2 | - | - | 30.6 | - | 13.5 |
| COL-Lenis and Esparza (1998) | 2.1 | - | - | 31.3 | - | - | 49.8 | - | 16.7 |
| ECU-Cueva and Yepez (2009) | 15.5 | - | - | 46.4 | - | - | 20.6 | - | 17.6 |
| PRY-Yoffe de Quiroz (2005) | 1.3 | - | - | 27.5 | - | - | 43.8 | - | 27.5 |
| ARG-Meiss (2016) | - | 71.4 | - | - | - | 25.8 | - | - | 2.8 |
| COL-Ramiréz-Martinéz (2015) | - | 39.7 | - | - | - | 56.8 | - | - | 3.5 |
| COL-González-Mariño (2005) | - | 39.0 | - | - | - | 57.2 | - | - | 3.7 |
| COL-Robledo-Abad (2005) | - | 55.5 | - | - | - | 40.5 | - | - | 3.9 |

| Country Code-Author (year) | Stage at diagnosis (%) | | | | | | | | |
|---|---|---|---|---|---|---|---|---|---|
| | I | I-IIa | I-II | II | I-III | IIb-III | III | III-IV | IV |
| PER-Infanzón (2000) | - | 61.4 | - | - | - | 34.6 | - | - | 4.0 |
| COL-Piñeros (2008) | - | 32.1 | - | - | - | 62.9 | | - | 5.0 |
| COL-García (2012) | - | 15.5 | - | - | - | 78.6 | - | - | 6.0 |
| COL-González-Mariño (2006) | - | 30.1 | - | - | - | 63.7 | - | - | 6.2 |
| COL-Angarita (2010) | - | 36.1 | - | - | - | 56.0 | - | - | 7.9 |
| COL-Pardo (2015) | - | 29.6 | - | - | - | 60.7 | - | - | 9.6 |
| ARG-Palazzo (2016) | - | 63.0 | - | - | - | 23.0 | - | - | 14.0 |
| COL-Martínez (2012) | - | 23.1 | - | - | - | 55.9 | - | - | 21.1 |
| COL-Pardo (2003) | - | 10.2 | - | - | - | 68.4 | - | - | 21.4 |
| CHL-Prieto (2011) (D) | - | - | 68.2 | - | - | - | 26.2 | - | 5.6 |
| CHL-Prieto (2011) (G) | - | - | 68.2 | - | - | - | 26.2 | - | 5.6 |
| CHL-Prieto (2011) (E) | - | - | 68.9 | - | - | - | 25.3 | - | 5.8 |
| CHL-Prieto (2011) (J) | - | - | 68.0 | - | - | - | 26.1 | - | 5.9 |
| CHL-Prieto (2011) (H) | - | - | 70.3 | - | - | - | 23.7 | - | 6.1 |
| CHL-Prieto (2011) (F) | - | - | 69.6 | - | - | - | 24.3 | - | 6.1 |
| CHL-Prieto (2011) (I) | - | - | 67.6 | - | - | - | 26.3 | - | 6.1 |
| CHL-Prieto (2011) (B) | - | - | 67.5 | - | - | - | 25.4 | - | 7.1 |
| GUF-Roué (2016) | - | - | 36.8 | - | - | - | 55.2 | - | 7.9 |
| CHL-Prieto (2011) (C) | - | - | 62.3 | - | - | - | 25.0 | - | 12.7 |
| CHL-Prieto (2011) (A) | - | - | 44.9 | - | - | - | 35.3 | - | 19.9 |
| CRI-Ortiz-Barboza (2011) | - | - | 35.4 | - | - | - | 38.6 | - | 26.1 |
| GUY-Taioli (2010) | - | - | - | - | 93.0 | - | - | - | 7.0 |
| SUR-van Leeuwaarde (2011) | - | - | 72.6 | - | - | - | - | 27.4 | - |
| BRA-Thuler and Mendonça (2005) (B) | - | - | 54.7 | - | - | - | - | 45.3 | - |
| PER-Díaz (1999) | - | - | 51.3 | - | - | - | - | 48.7 | - |
| BRA-Thuler and Mendonça (2005) (A) | - | - | 47.5 | - | - | - | - | 52.5 | - |

Percentage of T3/T4 cancers were used as proxy of stages III/IV for SUR-van Leeuwaarde (2011). Localized, regional and distant disease were considered as stages I-II, III and IV for CRI-Ortiz-Barboza (2011), CUB-Garrote (2011), and PRI-Ortiz (2010). Recruitment or diagnosis years: BHS-Mungrue (2016) A→C=2009→2011; BRA-Thuler and Mendonça (2005) A=1990-1994 B=1995-2002; CHL-Prieto (2011) A→J= 2000→2011; HND-Muñoz (2011) A=1999 B=2000-2004 C=2005-2009; MEX-Ortega-Cervantes

(2013) A→E=2006→2010; MEX-Arce-Salinas (2012) A=2008 B=2009. Study references are given on S3 File.
