# Supplementary material for: Stage at diagnosis and stage-specific survival of breast cancer in Latin America and the Caribbean: A systematic review and meta-analysis
Source: PLoS One. 2019 Oct 16;14(10):e0224012. doi: 10.1371/journal.pone.0224012 (PMC6799865; doi:10.1371/journal.pone.0224012)
Supplement: S6 Table — (PDF) [file pone.0224012.s010.pdf]

S6 Table. Characteristics of the studies included for the outcome survival probability

Country Code-Author (year)	Patients with breast cancer	Patients with known breast cancer stage (%)	Age*	Year of diagnosis	Sampling/ Study type	Country/ Province/ City	Location	Type of facility	Staging system	Staging method	Quality score
**Caribbean**											
CUB-Mora-Díaz (2004)	167	167 (100)	57.9 ± 14.1‡	1989-1998	Unclear/ Cohort	Havana	1 Center (Hospital Docente Julio Trigo López)	Public	TNM (UICC 4th ed.)	NR	9
CUB-Moreno de Miguel (1998)	145	145 (100)	NR	1985-1989	Unclear/ Cohort	Havana	1 Center (Instituto Nacional de Oncología y Radiobiología)	Public	TNM (5th ed.)	NR	4
CUB-Ricardo-Ramírez (2013)	132	132 (100)	NR	2002-2012	Unclear/ Cohort	Santiago de Cuba	1 Center (Hospital Provincial Docente Clinicoquirúrgico Saturnino Lora Torres)	Public	TNM	NR	6
CUB-González-Longoria Boada (2011)	171	170 (99.4)	58.2‡	1997-1998	Population-based/ Cohort	Granma province	Registro Nacional de Cáncer	Public	TNM (5th ed.)	NR	20
CUB-Garrote (2011)	2169	1772 (81.7)	NR	1994-1995	Population-based/ Cohort	Cuba	Registro Nacional de Cáncer	Public	TNM	NR	14
CUB-Fernández-Garrote (1998)	2375	2375 (100)	NR	1988-1989	Population-based/ Cohort	Cuba	Registro Nacional de Cáncer	Public	TNM	NR	17
HTI-DeGennaro (2018)	525	127 (24.2)	49.1	2013-/2017	Consecutive/ Cohort	Port-au-Prince	1 Center (started at Hospital Bernard Mevs then moved to St Luke's Hospital)	Private	TNM (6th ed.)	Clinical and imaging	15,5
**Central America**											

Country Code-Author (year)	Patients with breast cancer	Patients with known breast cancer stage (%)	Age*	Year of diagnosis	Sampling/ Study type	Country/ Province/ City	Location	Type of facility	Staging system	Staging method	Quality score
CRI-Quirós-Alpízar and Espinoza-Morales (2017)	221	221 (100)	NR	2006	Consecutive/ Cohort	San José	1 Center (Hospital San Juan de Dios)	Public	NR	NR	15,5
CRI-Ortiz-Barboza (2011)	2462	2105 (85.5)		1995-2000	Population-based/ Cohort	Costa Rica	National tumor registry	Public and private	TNM	NR	13
MEX-Flores-Luna (2008)	432	431 (99.8)	NR	1990-1999	Consecutive/ Cohort	Ciudad de México	1 Center (Servicio de Oncología del Hospital General de la Ciudad de México)	Public	TNM (5th ed.)	Clinical and imaging	16,5
MEX-Álvarez-Bañuelos (2016)	114	52 (45.6)		2009	Consecutive/ Cohort	Xalapa (estado de Veracruz)	1 Center (Centro Estatal de Cancerología)	Public	TNM (7th ed.)	NR	19,5
MEX-Ángeles-Llerenas (2016)	854	816 (95.6)	51 (IQR= 44.2-60.3)	2007-2009	Convenience/ Cohort	México	11 Centers	Public	TNM	NR	21,5
MEX-Lara-Medina (2011)	2074	2074 (100)	50 (19-96)	1998-2008	Consecutive/ Cohort	Ciudad de México	1 Center (Instituto Nacional de Cancerología)	Public	TNM (6th ed.)	NR	20,5
MEX-Reynoso-Noverón (2017)	4300	4300 (100)	NR	2007-2013	Consecutive/ Cohort	Ciudad de México	1 Center (Instituto Nacional de Cancerología)	Public	TNM	Clinical and imaging	21,5
MEX-di Filippo-Echeverri (2004)	111	49 (44.1)	(31-88)	1994-2002	Unclear/ Cohort	Ciudad de Mexico	1 Center (Hospital General de México)	Public	TNM	NR	11
MEX-Maffuz-Aziz (2016)	4902	3762 (76.7)	NR	2005-2014	Consecutive/ Cohort	Ciudad de México	1 Center (Instituto de Enfermedades de la Mama)	Public	TNM (7th ed.)	NR	10,5

Country Code-Author (year)	Patients with breast cancer	Patients with known breast cancer stage (%)	Age*	Year of diagnosis	Sampling/ Study type	Country/ Province/ City	Location	Type of facility	Staging system	Staging method	Quality score
MEX-Ramírez-Torres (2016)	120	120 (100)	≥40 <60†	2003-2010	Consecutive/ Cohort	Cuidad de Mexico	1 Center (Unidad Médica de Alta Especialidad del Hospital de Ginecología y Obstetricia n.◦. 3 Centro MédicoNacional La Raza, Instituto Mexicano del Seguro Social)	Public	TNM (6th ed.)	NR	17,5
MEX-Leon-Rodriguez (2017)	291	250 (85.9)	57 (27-89)	2000-2016	Consecutive/ Cohort	Ciudad de Mexico	1 Center (National Institute of Health Sciences and Nutrition Salvador Zubiran)	Public	TNM	NR	11,5
**South America**											
ARG-Iturbe (2011)	927	927 (100)	51 (28-92)	1978-2004	Consecutive/ Cohort	Argentina	6 centers (Grupo Oncologico Cooperativo del Sur)	NR	TNM (6th ed.)	Clinical and imaging	15,5
ARG-Arce (2013)	824	770 (93.4)	≥40 <60†	1994-2012	Consecutive/ Cohort	Posadas (povíncia de Misiones)	1 center (Sanatorio Boratti)	NR	TNM (6th ed.)	Clinical and imaging	18,5
ARG-Berra (2016)	131	131 (100)	≥40 <60†	2004-2014	Convenience/ Cohort	Mendoza	1 center (Servicio de Ginecología, Hospital Lagomaggiore)	Public	TNM	NR	15,5
BRA-Stival (2012)	345	333 (96.5)	≥40 <60†	1998-2002	Unclear/ Cohort	Goiânia	1 Center (Hospital Araújo Jorge da Associação de Combate ao Câncer de Goiás)	Private	TNM	NR	14
BRA-Ayala (2012)	655	655 (100)	55.1 ± 13.3‡	2000-2009	Convenience/ Cohort	Joinville	1 Center (Unidade de Especialidades Médicas do Sistema Único de Saúde)	Public	TNM (6th ed.)	NR	19,5

Country Code-Author (year)	Patients with breast cancer	Patients with known breast cancer stage (%)	Age*	Year of diagnosis	Sampling/ Study type	Country/ Province/ City	Location	Type of facility	Staging system	Staging method	Quality score
BRA-Guerra (2009)	745	726 (97.4)	56.0 (25-91)	1998-2000	Consecutive/ Cohort	Juiz de Fora	Multicentric	Public and private	TNMp (6th ed.)	NR	20,5
BRA-Schneider (2009)	1002	861 (85.9)	52 (13-89)	2000-2002	Consecutive/ Cohort	Florianópolis	2 Centers (Centro de Pesquisas Oncológicas de Santa Catarina; and Hospital de Caridade-Irmandade Nosso Senhor dos Passos)	Private	NR	NR	21,5
BRA-Moraes (2006)	252	252 (100)	54.0 (21-89)	1980-2000	Consecutive/ Cohort	Santa Maria	1 Center (Ambulatório de Mastologia do Hospital Universitário de Santa Maria)	Public	TNM (5th ed.)	Clinical	23,5
BRA-Vazquez (2016)	738	652 (88.3)	≥40 <60†	1985-2002	Consecutive/ Cohort	Barretos	1 Center (Barretos Cancer Hospital)	Private	TNM (6th ed.)	NR	20,5
BRA-Fayer (2016)	195	192 (98.5)	57	2000-2001	Consecutive/ Cohort	Juiz de Fora	1 Center	Public	TNM	NR	20,5
BRA-Carrara (2017)	98	98 (100)	48.5 ± 11.4‡	2005-2012	Consecutive/ Cohort	Barretos	1 Center (Hospital de Câncer de Barretos)	Private	TNM (7th ed.)	Clinical	17,5
CHL-Peralta (1995)	357	357 (100)	(22-92)	1985-1995	Consecutive/ Cohort	Región Metropolitana de Chile	Multicentric (Servicio de Salud Metropolitano Central)	Public	TNM	Clinical and imaging	10,5
CHL-Jürgensen (2009)	589	541 (91.6)	≥40 <60†	1972-2007	Consecutive/ Cohort	Santiago	1 Center (Hospital Clínico Fuerza Aérea de Chile)	Public	TNM	NR	10,5
CHL-Acevedo (2006)	501	420 (83.8)	53 (29-96)	1996-2005	Consecutive/ Cohort	Las Condes (Santiago)	1 Center (Centro Integral de la Mama de Clínica Las Condes)	NR	TNM (7th ed.)	NR	11,5

Country Code-Author (year)	Patients with breast cancer	Patients with known breast cancer stage (%)	Age*	Year of diagnosis	Sampling/ Study type	Country/ Province/ City	Location	Type of facility	Staging system	Staging method	Quality score
COL-Robledo-Abad (2005)	1328	1216 (91.6)	53 (25-92)	1989-2003	Consecutive/ Cohort	Bogotá	1 Center (Unidad Oncológica del Country)	Private	TNM (5th ed.)	NR	17,5
COL-Ospino (2010)	174	174 (100)	NR	2003-2004	Consecutive/ Cohort	Bogotá	1 Center (Instituto Nacional de Cancerología)	Public	TNM (6th ed.)	NR	23,5
COL-Ospino (2011)	75	75 (100)	NR	2003-2004	Consecutive/ Cohort	Bogotá	1 Center (Instituto Nacional de Cancerología)	Public	TNM (6th ed.)	NR	23,5
COL-Zuluaga-Liberato and Zuluaga-Cristancho (2016)	228	197 (86.4)	49.6	2005-2013	Consecutive/ Cohort	Bogotá	1 Center (consultorio médico especializado en oncología)	NR	TNM	NR	20,5
ECU-Jorge (1994)	21	21 (100)	(32-87)	1982-1992	Consecutive/ Cohort	Guayaquil	1 Center (Servicio de Oncología del Hospital Teodoro maldonado Carbo)	Public	TNM	NR	11,5
PER-Díaz (1999)	279	72 (25.8)	≥40 <60†	1966- 1995	Consecutive/ Cohort	Trujillo	1 Center (Hospital Belén)	Public	TNM (5th ed.)	Clinical and imaging	22,5
PER-Larrea-Fernández (2016)	75	75 (100)	42 (27-49)	2009-2010	Consecutive/ Cohort	Lima	1 Center (Hospital Nacional Guillermo Almenara Irigoyen)	Public	TNM (7th ed.)	NR	17,5
URY-Vázquez (2005)	1311	1185 (90.4)	61 (26-93)	1985-2003	Unclear/ Cohort	Montevideo	1 Center (Centro de Diagnóstico Mamario de la Asociación Española Primera de Socorros Mutuos)	Private	TNM	NR	13

Country Code-Author (year)	Patients with breast cancer	Patients with known breast cancer stage (%)	Age*	Year of diagnosis	Sampling/ Study type	Country/ Province/ City	Location	Type of facility	Staging system	Staging method	Quality score
VEN-Hung (2012)	312	312 (100)	≥40 <60†	2000-2008	Consecutive/ Cohort	La Yaguara	1 Center (Instituto de Oncología Dr. Miguel Pérez Carreño)	Public	NR	Clinical	20,5
VEN-Godoy (2000)	249	249 (100)	60 (27-88)	1988-1992	Consecutive/ Cohort	Caracas	1 Center (Instituto Oncológico Luis Razetti)	Public	TNM (4th ed.)	NR	12,5
VEN-Pacheco-Soler (2000)	143	139 (97.2)	76.3 (70-98)	1985-1993	Unclear/ Cohort	Caracas	1 Center (Hospital Oncológico Padre Machado)	Public	TNM (4th ed.)	NR	9
VEN-Ravelo-Celis and Ravelo Pagés (2007)	102	102 (100)	(27-90)	1981-1997	Consecutive/ Cohort	Caracas	1 Center (Instituto Diagnóstico)	Private	TNM	NR	8,5
VEN-Acosta-Marín (2011)	492	492 (100)	≥40 <60†	NR	Unclear/ Cohort	Caracas	1 Center (Centro Clínico de Estereotaxia)	Private	NR	NR	8
VEN-Vera (2002)	569	569 (100)	(23-81)	1978-1998	Consecutive/ Cohort	Caracas	1 Center (Servicio de Radioterapia Oncológica del Instituto Médico La Floresta)	Private	TNM	Clinical and imaging	10,5

ed., Edition; NR, Not reported; TNM, tumor, Lymph Node, Metastasis staging system; UICC, Union for International Cancer Control
* Age is given as median (range) unless otherwise indicated.
‡ mean age ± standard deviation.
† studies provided age as the distribution among age groups; the indicated age group includes most participants.
Study references are given on S3 File.
