# Supplementary material for: Stage at diagnosis and stage-specific survival of breast cancer in Latin America and the Caribbean: A systematic review and meta-analysis
Source: PLoS One. 2019 Oct 16;14(10):e0224012. doi: 10.1371/journal.pone.0224012 (PMC6799865; doi:10.1371/journal.pone.0224012)
Supplement: S7 Table — (PDF) [file pone.0224012.s011.pdf]

S7 Table. Survival results of the included studies

Country Code-Author (year)	Year of diagnosis	Study type and length of follow-up	Study description	Survival analysis type (start point, cause of death)	Tumor grade, hormone receptors and HER-2 positivity	Population and stage at diagnosis*	Stage-specific survival (OS, DSS, unclear)
**Caribbean**							
CUB-Mora-Diaz (2004)	1989-1998	Single-center retrospective	Clinical and therapeutic characterization and survival of early stages breast cancer	Kaplan-Meier (diagnosis, unclear)	HR+: NR<br>HER-2+: NR	167<br>Stage I: 52<br>Stage II: 115	5-year:<br>Stage I: 90<br>Stage II:82<br>12-year:<br>Stage I: 82<br>Stage II: 54<br>(unclear)
CUB-Moreno de Miguel (1998)	1985-1989	Single-center retrospective	Clinical characterization and survival of early stages breast cancer patients (<65 yo) who underwent conservative surgery followed by radiotherapy and chemotherapy	Not reported (unclear, unclear)	Grade III: NR<br>HR+: NR<br>HER-2+: NR	145<br>Stage I: 74<br>Stage IIa: 56<br>Stage IIb: 15	5-year:<br>Stage I: 98.5<br>Stage IIa: 94.1<br>Stage IIb: 85.6<br>12-year:<br>Stage I: 83.9<br>Stage IIa: 77.7<br>Stage IIb: 72.4<br>(unclear)
CUB-Ricardo-Ramirez (2013)	2002-2012	Single-center retrospective	Histopathologic characterization and survival of early breast cancer	Kaplan-Meier (unclear, unclear)	Grade III: NR<br>HR+: NR<br>HER-2+: NR	132	10-year:<br>Stage I: 92.5<br>Stage IIa: 77.6<br>Stage IIb: 54.7<br>(unclear)
CUB-Gonzáles-Longoria Boada (2011)	1997-1998	Population-based	Prognostic factors and survival analysis of breast cancer	Kaplan-Meier (diagnosis, cancer-specific)	Grade III: NR<br>HR+: NR<br>HER-2+: NR	170<br>Stage I: 12<br>Stage IIa: 46<br>Stage IIb: 34<br>Stage IIIa: 28<br>Stage IIIb: 45<br>Stage IV: 5	2-year:<br>Stage I: 91.7<br>Stage IIa: 93.5<br>Stage IIb: 73.5<br>Stage II: 86.3<br>Stage IIIa: 60.7<br>Stage IIIb: 40<br>Stage III: 49.3<br>Stage IV: 0.0<br>5-year:<br>Stage I: 83.3<br>Stage IIa: 78.3<br>Stage IIb: 61.8<br>Stage II: 52.3<br>Stage IIIa: 32.1<br>Stage IIIb: 31.1<br>Stage III: 31.5<br>Stage IV: 0.0

Country Code-Author (year)	Year of diagnosis	Study type and length of follow-up	Study description	Survival analysis type (start point, cause of death)	Tumor grade, hormone receptors and HER-2 positivity	Population and stage at diagnosis*	Stage-specific survival (OS, DSS, unclear)
							(DDS)
CUB-Garrote (2011)	1994-1995	Population-based retrospective Median follow-up: 54 months	Survival analysis of breast cancer	Actuarial (diagnosis, unclear)	Grade III: NR<br>HR+: NR<br>HER-2+: NR	Localized: 944<br>Regional: 722<br>Distant: 106	5-year:<br>localized: 81.6<br>regional: 58.9<br>distant: 30.2
CUB-Fernández-Garrote (1998)	1988-1989	Population-based retrospective	Survival analysis of breast cancer	Actuarial (diagnosis, unclear)	Grade III: NR<br>HR+: NR<br>HER-2+: NR	2375	5-year:<br>localized: 70.9<br>regional: 46.4<br>distant: 20.4
HTI-DeGennaro (2018)	2013-/2017	Single-center retrospective	Epidemiological, clinical, and histopathological characterization of breast cancer	Unclear (date of first consultation, unclear)	Grade III: 29/2018 (13.3%)<br>ER+: 127/245 (51.8%)<br>HER-2+: 35/179 (19.6%)	525 (all stages)	1-year:<br>Stage IV: 73.5<br>2-year:<br>Stage IV: 28.2
**Central America**							
CRI-Quirós-Alpízar (2017)	2006	Single-center retrospective	Histopathologic characterization and survival analysis of breast cancer (<50 yo)	Kaplan-Meier (unclear, unclear)	Grade III: 128/188 (68.0%)<br>ER+: 158/219 (72.1%)<br>PR+: 135/221 (61.6%)<br>HER-2+: 35/221 (16.0%)	221 (all stages)	5-year:<br>Stage I: 96.0 (74.8-99.4)<br>Stage II: 82.6 (69.3-90.6)<br>Stage III: 87.5 (70.1-95.1)
CRI-Ortiz-Barbosa (2011)	1995-2000	Population-based Median follow-up: 45 months	Survival analysis of breast cancer	Actuarial (diagnosis, unclear)	Grade III: NR<br>HR+: NR<br>HER-2+: NR	2462 (all stages)	5-year:<br>Localized: 89.9<br>Regional: 77.1<br>Distant: 3
MEX-Flores-Luna (2008)	1990-1999	Single-center retrospective	Prognostic factors and survival analysis of breast cancer	Kaplan-Meier (diagnosis, cancer-specific)	Grade III: 8/432 (1.9%)<br>HR+: NR<br>HER-2+: NR	432<br>Stage I: 42<br>Stage IIa: 98<br>Stage IIb: 129<br>Stage IIIa: 71<br>Stage IIIb: 79<br>Stage IV: 12	5-year:<br>Stage I: 82<br>Stage IIa: 65.3<br>Stage IIb: 70.4<br>Stage IIIa: 44.2<br>Stage IIIb: 47.5<br>Stage IV: 15
MEX-Álvarez-Bañuelos (2016)	2009	Single-center retrospective	Prognostic factors and survival analysis of breast cancer	Kaplan-Meier (diagnosis, cancer-specific)	Grade III: 43/80 (53.8%)<br>ER+: 47/70 (67.1%)<br>PR+: 32/65 (49.2%)<br>HER-2+: 24/74 (32.4%)	52	5-year:<br>Stage IIIb: 38<br>Stage IV: 10

Country Code-Author (year)	Year of diagnosis	Study type and length of follow-up	Study description	Survival analysis type (start point, cause of death)	Tumor grade, hormone receptors and HER-2 positivity	Population and stage at diagnosis*	Stage-specific survival (OS, DSS, unclear)
MEX-Ángeles-Llerenas (2016)	2007-2009	11 centers prospective	Analysis care-delivery delays on survival among women with breast cancer	Kaplan-Meier (start of treatment, cancer-specific)	Grade III: NR<br>HR+: NR<br>HER-2+: NR	854<br>Stages I-IIa: 281<br>Stages IIb-IV: 553	5-year:<br>Stage I-IIa: 92.1 (87.8-95.0)<br>Stage IIb-IV: 69.8 (63.2-75.4)
MEX-Lara-Medina (2011)	1998-2008	Single-center retrospective Median follow-up: 17 months	Prevalence and survival analysis of triple-negative breast cancer (survival is given for all histological types)	Kaplan-Meier (diagnosis, cancer-specific)	Grade III: 1054/2074 (50.8%)<br>HR+: 1172/2074 (56.5%)<br>HER-2+: 423/2074 (20.4%)	2074<br>Stage I: 201<br>Stage II: 718<br>Stage III: 913<br>Stage IV: 243	5-year:<br>Stage I: 94.20 (88.91-99.49)<br>Stage II: 89.90 (84.81-94.99)<br>Stage III: 73.80 (67.53-80.07)<br>Stage IV: 33.80 (32.24-35.36)
MEX-Reynoso-Noverón (2017)	2007-2013	Single-center retrospective Median follow-up: 40.5 months	Survival analysis of breast cancer patients from Seguro Popular	Kaplan-Meier (diagnosis, all-cause)	Grade III: 2063/4300 (51.3%)<br>HR+: NR<br>HER-2+: 979/4300 (23.2%)	4300	5-year:<br>Stages I-IIa: 97 (95-98)<br>Stages IIb-IIIc: 82 (80-84)<br>Stage IV: 36 (30-42)
MEX-Di Filippo-Echeverri (2004)	1994-2002	Single-center retrospective Mean follow-up: 45.75±18.4 months	Survival analysis of early stage breast cancer patients who underwent conservative surgery	Kaplan-Meier (diagnosis, unclear)	Grade III: 19/98 (19.4%)<br>HR+: NR<br>HER-2+: NR	111	5-year:<br>Stages I-II: 87
MEX-Maffuz-Aziz (2016)	2005-2014	Single-center retrospective Mean follow-up: 28 (1-266) months	Global and disease-free survival analysis of patients with breast cancer analyzed by risk groups	Kaplan-Meier (unclear, unclear)	Grade III: NR<br>HR+: NR<br>HER-2+: NR	3762	5-year:<br>Stage I-IIa: 96.8 ±0.6<br>Stage IIb-IIIc: 74.6 ±1.7<br>Stage IV: 35.9 ±5.1
MEX-Ramirez-Torres (2016)	2003-2010	Single-center retrospective Median follow-up: 53 (12-85) months	Survival analysis of patients with advanced breast cancer treated with high doses of neoadjuvant epirubicin and different periods	Kaplan-Meier (diagnosis, all-cause)	Grade III: 79/120 (65.8%)<br>ER+: 80/120 (66.7%)<br>PR+: 68/120 (56.7%)<br>HER-2+: 31/120 (25.8%)	120<br>Stage IIIa: 76<br>Stage IIIb-IIIc: 44	5-year:<br>Stage IIIa: 80<br>Stages IIIb-IIIc: 58
MEX-Leon-Rodriguez (2017)	2000-2016	Single-center retrospective Median follow-up: 40 months	Assessment of treatment intervals in breast cancer patients according to the detection method (breast self-examination vs. screening)	Kaplan-Meier (unclear, unclear)	Grade III: NR<br>HR+: NR<br>HER-2+: NR	291<br>Selfexam:<br>Stage I-IIa: 85<br>Stage IIb-IIIc: 65<br>Screening:<br>Stages I-IIa: 80<br>Stages IIb-IIIc: 6	40-month:<br>Selfexam:<br>Stages I-IIa: 93<br>Stages IIb-IIIc: 65<br>Screening:<br>Stages I-IIa: 97.5<br>Stages IIb-IIIc:100
**South America**							

Country Code-Author (year)	Year of diagnosis	Study type and length of follow-up	Study description	Survival analysis type (start point, cause of death)	Tumor grade, hormone receptors and HER-2 positivity	Population and stage at diagnosis*	Stage-specific survival (OS, DSS, unclear)
ARG-Iturbe (2011)	1978-2004	6 centers retrospective<br>Median follow-up: 8.4 (0.3-30) years	Report the incidence of local and distant recurrence, disease free interval and OS in patients with stage I and stage II breast cancer over a period of 26 years	Kaplan-Meier (diagnosis, all-cause)	Grade III: NR<br>ER+: 460/628 (73.2%)<br>PR+: 366/530 (69.1%)<br>HER-2+: NR	927<br>Stage I: 350<br>Stage II: 577	5-year: 82<br>10-years: 62<br>15 years: 49<br>20 years: 39<br>25 years: 28
ARG-Arce (2013)	1994-2012	Single-center retrospective<br>Median follow-up: 50,5 (1-218) months	Analysis of 1000 consecutive patients diagnosed and operated on for breast over a period of 18 years	Kaplan-Meier (unclear, all-cause)	Grade III: NR<br>HR+: NR<br>HER-2+: NR	770<br>Stage I: 213<br>Stage: IIa: 207<br>Stage IIb: 170<br>Stage IIIa: 99<br>Stage IIIb: 41<br>Stage IIIc: 40	18-year:<br>Stage I: 88.3<br>Stage IIa: 84.5<br>Stage IIb: 72.4<br>Stage IIIa: 61.6<br>Stage IIIb: 56.1<br>Stage IIIc: 55
ARG-Berra (2016)	2004-2014	Single-center retrospective	Assessment of the influence of the immunohistochemical profile on disease-free survival and global survival in locally advanced breast cancer	Kaplan-Meier (diagnosis, unclear)	HER-2+: 15<br>Triple-negative: 23<br>Grade III: NR<br>HR+: NR<br>HER-2+: 15/131 (11.5%)	131 (Stages IIb-IIIb)<br>Luminal A: 70<br>Luminal B: 23<br>HER-2+: 15	2-year: Luminal A: 95.28<br>Luminal B: 88.14<br>HER-2+: 82.9<br>Triple-negative: 75.7<br>4-year: Luminal A: 78.56<br>Luminal B: 61.7<br>HER-2+:20.1<br>Triple-negative: 51.2<br>6-year: Luminal A: 60.23<br>Luminal B: 52.9<br>HER-2+: NR<br>Triple-negative: 37<br>10-year: Luminal A: 36.97<br>Luminal B: NR<br>HER-2+: NR<br>Triple-negative: 18
BRA-Stival (2012)	1998-2002	Single-center retrospective	Assess the clinical and pathological aspects of breast cancer patients to compare the impact of triple-negative phenotype prognosis	Kaplan-Meier (diagnosis, all-cause)	Grade III: NR<br>HR+: NR<br>HER-2+: NR	333<br>Stage I: 31<br>Stage II: 182<br>Stage III: 101<br>Stage IV: 19	5-year:<br>Stage I: 93.5<br>Stage II: 75.8<br>Stage III: 43.6<br>Stage IV: 31.6
BRA-Ayala (2012)	2000-2009	Single-center retrospective	Survival analysis according to stage at diagnosis	Kaplan-Meier (diagnosis, cancer-specific)	Grade III: NR<br>HR+: NR<br>HER-2+: NR	655<br>Stage I: 131<br>Stage II: 315<br>Stage III: 178<br>Stage IV: 31	5-year:<br>Stage I: 97<br>Stage II: 88<br>Stage III: 51<br>Stage IV: 17

Country Code-Author (year)	Year of diagnosis	Study type and length of follow-up	Study description	Survival analysis type (start point, cause of death)	Tumor grade, hormone receptors and HER-2 positivity	Population and stage at diagnosis*	Stage-specific survival (OS, DSS, unclear)
BRA-Guerra (2009)	1998-2000	Multicentric retrospective	Analysis of five-year survival and the main prognostic factors among women with invasive breast cancer that had undergone surgical treatment	Kaplan-Meier (diagnosis, cancer-specific)	Grade III: NR<br>HR+: NR<br>HER-2+: NR	745<br>Stage I: 125<br>Stage II: 343<br>Stage III: 227<br>Stage IV: 31	5-year:<br>Stage I: 92.7<br>Stage II: 88.3<br>Stage III: 67<br>Stage IV: 54
BRA-Schneider (2009)	2000-2002	2 centers retrospective	Analysis of breast cancer survival and associated factors, based on a historical cohort of women with breast cancer diagnosis	Kaplan-Meier (diagnosis, cancer-specific)	Grade III: NR<br>HR+: NR<br>HER-2+: NR	861	5-year:<br>Stage I: 93.6 (89.8-97.5)<br>Stage II: 87.8 (84.6-91.1)<br>Stage III: 62.5 (56.3-69.5)<br>Stage IV: 27.3 (19.6-38.1)
BRA-Moraes (2006)	1980-2000	Single-center retrospective<br>Median follow-up: 62 (0,4-241) months	Description of health conditions and to estimate the survival of patients diagnosed with breast cancer	Kaplan-Meier (date of surgery, cancer-specific)	Grade III: 18/252 (9.6%)<br>ER+: 119/171 (69.6%)<br>PR+: 21/39 (53.8%)<br>HER-2+: NR	252	5-year: 87.7<br>Stage I: 97<br>Stage IIa: 96<br>Stage IIb: 90<br>Stage IIIa-IIIb: 73<br>Stage IV: 57
BRA-Vazquez (2016)	1985-2002	Single-center retrospective<br>Mean follow-up: 6.1 (0-22.6) months	Comparison of socio-demographic, clinical and pathological characteristics with long-term survival, between young (≤40 yo) and older (50-69 yo) patients with breast cancer	Kaplan-Meier (diagnosis, all-cause)	Grade III: NR<br>HR+: NR<br>HER-2+: NR	652<br>≤40 years: 319<br>Stage I: 38<br>Stage II: 136<br>Stage III 101<br>Stage IV: 44<br>50-69 years: 333<br>Stage I: 37<br>Stage II: 135<br>Stage III: 122<br>Stage IV: 39	10-year:<br>≤40 years:<br>Stage I: 90.9<br>Stage II: 66.2<br>Stage III: 20.4<br>Stage IV; 0.049<br>50-69 years:<br>Stage I: 66.8<br>Stage II: 67.6<br>Stage III: 28.3<br>Stage IV: 0
BRA-Fayer (2016)	2000-2001	Single-center retrospective	Analysis of ten-year survival and prognostic factors in women with invasive breast cancer	Kaplan-Meier (diagnosis, all-cause)	Grade III: NR<br>ER+: 109/146 (74.7%)<br>PR+: 101/145 (69.7%)<br>HER-2+: 54/127 (42.5%)	192 (all stages)	10-year:<br>Stage I: 75.1 (57.1-86.3)<br>Stage II: 63 (51.7-72.4)<br>Stage III: 26.3 (12.9-41.8)<br>Stage IV: 14.3 (0.71-46.5)
BRA-Carrara (2017)	2005-2012	Single-center retrospective<br>Mean follow-up: 64.1 (13.4-105.7) months	Assessment of ipsilateral breast tumor recurrence after breast-conserving surgery for locally advanced breast cancer	Actuarial (unclear, unclear)	Grade III: 40/98 (41.7%)<br>ER+: 53/98 (54.1%)<br>PR+: 46/98 (46.9%)<br>HER-2+: NR	98<br>Stage IIb: 13 (13.3)<br>Stage III: 85 (86.7)	5-year: 81.2<br>8-year: 71.4

Country Code-Author (year)	Year of diagnosis	Study type and length of follow-up	Study description	Survival analysis type (start point, cause of death)	Tumor grade, hormone receptors and HER-2 positivity	Population and stage at diagnosis*	Stage-specific survival (OS, DSS, unclear)
CHL-Peralta (1995)	1985-1995	Multicentric<br>Median follow-up: 47.3 months	Clinical and pathological assessment and survival analysis of breast cancer patients	Actuarial (unclear, unclear)	Grade III: NR<br>HR+: 150/208 (72.1%)<br>HER-2+: NR	357 (all stages)	5-year:<br>Stage IV: 9.3<br>8-year:<br>Stage I: 100<br>Stage II 57.1<br>Stage III: 42.9
CHL-Jurgensen (2009)	1972-2007	Single-center retrospective	Establish if the presence of multifocal-multicentric breast cancer, in comparison to the unifocal breast cancer, is associated to worse prognosis	Kaplan-Meier (unclear, unclear)	Grade III: NR<br>HR+: NR<br>HER-2+: NR	541 (underwent surgery)<br>Stage I: 151<br>Stage II: 233<br>Stage III: 103<br>Stage IV: 54	5-year:<br>Stage I: 89.4<br>Stage II: 75.5<br>Stage III: 55.3<br>Stage IV: 33.3
CHL-Acevedo (2006)	1996-2005	Single-center retrospective<br>Median follow-up: 63 (3-120) months	Clinical and pathological assessment and survival analysis of breast cancer patients	Kaplan-Meier (unclear, unclear)	Grade III: NR<br>HR+: NR<br>HER-2+: NR	420<br>Stage I: 145<br>Stage II: 210<br>Stage III: 55<br>Stage IV: 10	5-year:<br>Stage I: 99.3<br>Stage II: 93.3<br>Stage III: 92.9<br>Stage IV: 63.9
COL-Robledo-Abad (2005)	1989-2003	Single-center retrospective<br>Mean follow-up: 43.9 (1-173) months	Assessment of characteristics and survival of patients with of breast carcinoma	Kaplan-Meier (diagnosis, all-cause)	Grade III: NR<br>HR+: 771/982 (78.5%)<br>HER-2+: NR	1216	5-year:<br>Stage I: 94.4<br>Stage IIa: 88.2<br>Stage IIb: 85.1<br>Stage III: 63.1<br>Stage IV: 33.4<br>8-year:<br>Stage I: 91.9<br>Stage IIa: 83.1<br>Stage IIb: 71.5<br>Stage III: 56.4<br>Stage IV: 22.3
COL-Ospino (2010)	2003-2004	Single-center retrospective	Analysis of survival after treatment with mastectomy and radiotherapy with curative purposes for locally advance breast cancer	Kaplan-Meier (end of treatment, all-cause)	Grade III: 46/163 (28.2%)<br>ER+: 108/168 (64.3%)<br>PR+: 111/169 (65.7%)<br>HER-2+: 17/28 (60.7%)	174	5-year:<br>Locally advanced: 84.4
COL-Ospino (2011)	2003-2004	Single-center retrospective	Assessment of the results of patients with early breast cancer treated with	Kaplan-Meier (end of treatment, all-cause)	Grade III: 11/69 (15.9%)<br>ER+: 52/71 (73.2%)<br>PR+: 52/71 (73.2%)<br>HER-2+: 2/6 (33.3%)	75	5-year:<br>Early stage: 92.9 (84.7-100)

Country Code-Author (year)	Year of diagnosis	Study type and length of follow-up	Study description	Survival analysis type (start point, cause of death)	Tumor grade, hormone receptors and HER-2 positivity	Population and stage at diagnosis*	Stage-specific survival (OS, DSS, unclear)
			conservative surgery and radiotherapy				
COL-Zuluaga-Liberato (2016)	2005-2013	Single-center retrospective<br>Median follow-up: 49.6 (2-116) months	Histopathological and survival analysis of breast cancer patients	Kaplan-Meier (unclear, all-cause)	Grade III: 69/223 (30.9%)<br>ER+: 206/225 (91.6%)<br>PR+: 179/225 (79.6%)<br>HER-2+: NR	197<br>Stage II: 144<br>Stage III: 47<br>Stage IV: 6	5-year:<br>Stage II: 85.9<br>Stage III: 66.9<br>Stage IV: 50
ECU-Jorge (1994)	1982-1992	Single-center retrospective<br>Range of follow-up: 6-126 months	Assessment of the clinical evolution of patients diagnosed with breast cancer	Actuarial (unclear, unclear)	Grade III: NR<br>HR+: NR<br>HER-2+: NR	21	48 months:<br>Locally advanced: 5
PER-Diaz (1999)	1966- 1995	Single-center retrospective	Comparison of the long - term surgical rates according to different clinical-pathologic and therapeutic factors, and the 10- year surgical and recurrence rates after classic radical mastectomy	Kaplan-Meier (surgery, cancer-specific)	Grade III: NR<br>HR+: NR<br>HER-2+: NR	72	5-year:<br>Stages I-II: 71<br>12-year:<br>Stages I-II: 59
PER-Larea-Fernández (2016)	2009-2010	Single-center retrospective<br>Median follow-up: 27.7 months	Description of the main clinical and pathological features, subtypes of breast cancer by immunohistochemistry, most frequently stage and survival rates in women (<50 yo)	Kaplan-Meier (date of hospital admission, unclear)	Grade III: 46/75 (61.3%)<br>ER+: 45/75 (60.0%)<br>PR+: 45/75 (60.0%)<br>HER-2+: 22/75 (29.3%)	75<br>Stage I: 11<br>Stage II: 36<br>Stage III: 26<br>Stage IV: 2	3-year:<br>Stage I: 100<br>Stage II: 89.3<br>Stage III: 74.7<br>Stage IV: 57.8
URY-Vázquez (2005)	1985-2003	Single-center retrospective<br>Median follow-up: 72.2 months	Characterization and survival analysis of breast cancer patients	Kaplan-Meier (unclear, all-cause)	Grade III: NR<br>ER+: 362/552 (65.6%)<br>PR+: 290/552 (52.5%)<br>HER-2+: NR	1185<br>Stage I: 415<br>Stage II: 529<br>Stage III: 225<br>Stage IV: 16	5-year:<br>Stage I: 98 (97-99)<br>Stage II: 88 (85-91)<br>Stage III: 79 (73-85)<br>Stage IV: 40 (13-67)<br>10-year:<br>Stage I: 97 (95-99)<br>Stage II: 79 (75-83)<br>Stage III: 65 (57-73)<br>Stage IV: 30 (4-56)

Country Code-Author (year)	Year of diagnosis	Study type and length of follow-up	Study description	Survival analysis type (start point, cause of death)	Tumor grade, hormone receptors and HER-2 positivity	Population and stage at diagnosis*	Stage-specific survival (OS, DSS, unclear)
VEN-Hung (2012)	2000-2008	Single-center retrospective	Determination if the proliferation index based on the Ki-67 antigen's expression is a prognostic factor in the molecular classes of the breast carcinoma	Kaplan-Meier (diagnosis, cancer-specific)	Grade III: 99/312 (31.7%)<br>HR+: NR<br>HER-2+: NR	312 (ductal carcinoma)	5-year:<br>Stage I: 52.7 (45.9-59.6)<br>Stage II: 53.0 (50.4-55.7)<br>Stage III: 46.2 (43.3-49.1)<br>Stage IV: 27.8 (20.0-35.7)
VEN-Godoy (2000)	1988-1992	Single-center retrospective<br>Mean follow-up: 63 (6-124) months	Assessment of the incidence, prognosis and treatment of stage III breast carcinoma	Kaplan-Meier (unclear, unclear)	Grade III: 91/249 (37.0%)<br>HR+: 74/97 (76.3%)<br>HER-2+: NR	249	5-year:<br>Stages IIIa-IIIb: 67
VEN-Pacheco-Soler (2000)	1985-1993	Single-center retrospective<br>Mean follow-up: 35.65 (1-120) months	Characterization and survival analysis of elderly breast cancer patients (>70 yo)	Actuarial (unclear, unclear)	Grade III: NR<br>HR+: NR<br>HER-2+: NR	139	5-year:<br>Stage I: 100<br>Stage IIa: 76<br>Stage IIb: 70<br>Stage IIIa: 44<br>Stage IIIb: 48<br>Stage IV: 46
VEN-Ravelo-Celis (2007)	1981-1997	Single-center<br>Mean follow-up: 54 months	Describe the experience of one care center with stage I-II breast cancer	Actuarial (unclear, unclear)	Grade III: NR<br>ER+: 52/74 (70.3%)<br>HER-2+: NR	102	5-year:<br>Stage I: 93<br>Stage IIa: 82<br>Stage IIb: 60<br>Stage IIIa: 56
VEN-Acosta-Marín (2011)	NR	Single-center retrospective<br>Mean follow-up: 5 years	Comparison of infiltrante lobulillar carcinoma and ductal carcinoma in long-term follow up by stage surgical treatment, neoadyuvant, adjuvant therapy	Kaplan-Meier (unclear, unclear)	Grade III: NR<br>HR+: NR<br>HER-2+: NR	492 (ductal or lobular)	5-year:<br>Lobular<br>Stage I: 96.3<br>Stage II: 94.4<br>Stage III: 90.5<br>Ductal<br>Stage I: 90.5<br>Stage II: 88.4<br>Stage III: 83.2<br>10-year:<br>Lobular<br>Stage I: 94.3<br>Stage II: 91<br>Stage III: 88.6<br>Ductal<br>Stage I: 88.6<br>Stage II: 85.3<br>Stage III: 81.8<br>12-year:

Country Code-Author (year)	Year of diagnosis	Study type and length of follow-up	Study description	Survival analysis type (start point, cause of death)	Tumor grade, hormone receptors and HER-2 positivity	Population and stage at diagnosis*	Stage-specific survival (OS, DSS, unclear)
							Lobular<br>Stage: 92.5<br>Stage II: 88<br>Stage III: 81.3<br>Ductal<br>Stage I: 87<br>Stage II: 82.4<br>Stage III: 74.9
VEN-Vera (2002)	1978-1998	Single-center retrospective<br>Mean follow-up: 62 months	Analysis of the results of the breast cancer conservative treatment followed by postoperative radiotherapy	Actuarial (unclear, unclear)	Grade III: NR<br>HR+: NR<br>HER-2+: NR	569 (adjuvant radiotherapy)	5-year:<br>Stage Ia: 89<br>Stage Ib: 82<br>Stage IIa: 88<br>Stage IIb: 78<br>10-year:<br>Stage Ia: 81<br>Stage Ib: 63<br>Stage IIa: 73<br>Stage IIb: 56

DDS: Disease specific survival; IQR: Interquartile range; OS: Overall survival; unclear: study author did not clearly state what causes of death were considered for survival analysis

* Reference values for Grade III, HR, ER, PR, and HER-2 may be greater than the number of staged patients because it refers to the study population with information for these parameters. ER+: Estrogen receptor positive. HR+: Hormone receptor positive. HER-2+: Human Epidermal growth factor Receptor 2 positive. PG+: Progesterone receptor positive. Study references are given on S3 File.
